# Effect of ATG12–ATG5-ATG16L1 autophagy E3-like complex on the ability of LC3/GABARAP proteins to induce vesicle tethering and fusion

**DOI:** 10.1007/s00018-023-04704-z

**Published:** 2023-02-02

**Authors:** Marina N. Iriondo, Asier Etxaniz, Yaiza R. Varela, Uxue Ballesteros, Melisa Lázaro, Mikel Valle, Dorotea Fracchiolla, Sascha Martens, L. Ruth Montes, Félix M. Goñi, Alicia Alonso

**Affiliations:** 1 Instituto Biofisika (UPV/EHU, CSIC), University of the Basque Country, 48940 Leioa, Spain; 2 Department of Biochemistry and Molecular Biology, University of the Basque Country, 48940 Leioa, Spain; 3 CIC bioGUNE, Basque Research and Technology Alliance (BRTA), Bizkaia Technology Park, Building 800, 48160 Derio, Bizkaia Spain; 4 Max Perutz Labs, University of Vienna, Vienna BioCenter, Dr. Bohr-Gasse 9, 1030 Vienna, Austria

**Keywords:** Autophagosome expansion, Human ATG8, ATG12 UBL system, Membrane fusion, Autophagy conjugation systems, Lipid-protein interaction

## Abstract

In macroautophagy, the autophagosome (AP) engulfs portions of cytoplasm to allow their lysosomal degradation. AP formation in humans requires the concerted action of the ATG12 and LC3/GABARAP conjugation systems. The ATG12–ATG5-ATG16L1 or E3-like complex (E3 for short) acts as a ubiquitin-like E3 enzyme, promoting LC3/GABARAP proteins anchoring to the AP membrane. Their role in the AP expansion process is still unclear, in part because there are no studies comparing six LC3/GABARAP family member roles under the same conditions, and also because the full human E3 was only recently available. In the present study, the lipidation of six members of the LC3/GABARAP family has been reconstituted in the presence and absence of E3, and the mechanisms by which E3 and LC3/GABARAP proteins participate in vesicle tethering and fusion have been investigated. In the absence of E3, GABARAP and GABARAPL1 showed the highest activities. Differences found within LC3/GABARAP proteins suggest the existence of a lipidation threshold, lower for the GABARAP subfamily, as a requisite for tethering and inter-vesicular lipid mixing. E3 increases and speeds up lipidation and LC3/GABARAP-promoted tethering. However, E3 hampers LC3/GABARAP capacity to induce inter-vesicular lipid mixing or subsequent fusion, presumably through the formation of a rigid scaffold on the vesicle surface. Our results suggest a model of AP expansion in which the growing regions would be areas where the LC3/GABARAP proteins involved should be susceptible to lipidation in the absence of E3, or else a regulatory mechanism would allow vesicle incorporation and phagophore growth when E3 is present.

**Supplementary Information:**

The online version contains supplementary material available at 10.1007/s00018-023-04704-z.

## Introduction

Autophagy is a highly conserved degradation pathway that is essential for eukaryotic cell homeostasis and health [1]. Among the various types of autophagy [2], macroautophagy (hereafter autophagy) is the best characterized one. Its activation is followed by the formation of a nascent double membrane structure, the phagophore, which develops into the so-called autophagosome (AP). The AP is capable of engulfing portions of the cytoplasm, then fusing with lysosomes/vacuoles, where the sequestered cargo is degraded and recycled [3]. When autophagy is activated under starvation conditions, it ensures cell survival by providing nutrients (non-selective autophagy). Moreover, it can also play a housekeeping role, selectively removing misfolded or aggregated proteins, damaged and/or superfluous organelles, as well as intracellular pathogens (selective autophagy) [4]. Altered autophagy mechanisms can give rise to a whole range of diseases, including cancer and neurodegeneration [5].

To date, more than 40 Atg proteins involved in AP biogenesis have been reported [6], including two ubiquitin-like (UBL) conjugation systems. Both are interconnected and need to act together for a proper AP assembly in vivo [7–9]. In humans, the first UBL system, the ATG12 system, is composed of ATG5, ATG12, ATG10, ATG7 and ATG16L1 proteins [10]. At the beginning of AP formation, ATG12 is activated by the E1-like enzyme ATG7. Then, the activated ATG12 is transferred to the cysteine residue of ATG10 (E2-like enzyme) and covalently conjugated to ATG5 via the formation of an isopeptide bond with a lysine residue. The resulting ATG12–ATG5 conjugate interacts with ATG16L1 to form the ATG12–ATG5-ATG16L1 complex, which acts as the E3 ligase enzyme of the second UBL conjugation system [11–14]. In the present work, the ATG12–ATG5-ATG16L1 complex is referred to as the E3-like complex (E3 for short).

The second UBL system is the LC3/GABARAP or human ATG8 lipidation system. It is composed of ATG4, ATG7, ATG3, and the LC3/GABARAP protein family members. In a first step, ATG4 promotes the exposure of LC3/GABARAP protein C-terminal glycine. Then, the LC3/GABARAP protein is activated by the same E1-like enzyme, ATG7, and transferred to a different E2-like enzyme, ATG3. ATG3, in collaboration with E3, directs LC3/GABARAP protein to the autophagosomal membrane and catalyzes the conjugation of LC3/GABARAP family members to phosphatidylethanolamine (PE) [15–17]. This covalent lipid-protein binding is known as lipidation and results in the anchoring of LC3/GABARAP proteins to autophagic membranes. The final product, LC3/GABARAP–PE, is considered as an autophagy marker in cells.

Although the cell can recognize the cargo to be degraded independently of LC3/GABARAP [18–20], in certain types of selective autophagy, LC3/GABARAP proteins are able to participate in cargo selection, interacting with different receptors [21, 22], which can be proteins[23, 24] or lipids [25–28]. In addition, once attached to the membrane, they are also involved in autophagosomal membrane expansion, closure, and fusion with lysosomes [29–33]. The human LC3/GABARAP family can be divided into two subfamilies: LC3A, LC3B and LC3C form the LC3 subfamily, while GABARAP, GABARAPL1 and GABARAPL2 (also known as GATE16) form the GABARAP subfamily [34]. The existence of at least six members of the LC3/GABARAP family in humans while only one, Atg8, is known in yeast suggests that each of them could play a different role in the autophagy process, cargo recognition during selective autophagy, and at different stages during AP formation.

The mode of participation of LC3/GABARAP in the phagophore expansion process to form the AP is still unclear. Some studies point to the hemifusion of vesicles into the growing phagophore [15, 33]. In this laboratory, Landajuela et al. [35] carried out the in vitro reconstitution of the lipidation process, in the absence of E3, for three of the LC3/GABARAP family members: LC3B, GABARAP and GABARAPL2. They found that lipidated forms of the GABARAP subfamily proteins promoted a more extensive membrane tethering and lipid mixing than LC3B. They also showed that negative curvature-inducing lipids (e.g., cardiolipin, diacylglycerol) facilitated the fusion process. Those results strongly supported the hypothesis of a highly bent structural fusion intermediate (stalk) during AP biogenesis and reinforced lipids as key regulators of autophagy [35, 36].

In vitro studies with yeast proteins have investigated the interplay between both ubiquitin-like systems and their interaction with membranes [11, 12, 37] showing that the presence of yeast E3 (Atg12–Atg5-Atg16), increased Atg3 activity, boosting the lipid-protein conjugation reaction and specifying the membrane site where Atg8 lipidation occurred. Studies with human proteins are scarce, as the full human E3 was only recently available, expressed in eukaryotic cells [38, 39]. In the present study, the lipidation of six members of the LC3/GABARAP family, namely LC3A, LC3B, LC3C, GABARAP, GABARAPL1 and GABARAPL2, has been reconstituted in the presence and absence of E3. Moreover, to shed light into the phagophore expansion process, the molecular mechanisms by which E3 and the different members of the LC3/GABARAP family interact in triggering vesicle tethering/aggregation and fusion have been investigated.

## Materials and methods

### Materials

L-α-phosphatidylcholine from hen egg yolk (ePC, 840051), 1,2-dioleoyl-sn-glycero-3-phosphatidylethanolamine (DOPE, 850725), liver phosphatidylinositol (PI, 840042), egg dioleoylglycerol (DOG, 800811), 1,2-dioleoyl-sn-glycero-3-phosphatidylethanolamine-N-lissamine rhodamine B sulfonyl (Rho-PE, 810150) and 1-oleoyl-2-{6-[(7-nitro-2–1,3-benzoxadiazol-4-yl)amino]hexanoyl}-sn-glycero-3-phosphoethanolamine (NBDtail-PE, 810155) were purchased from Avanti Polar Lipids, Inc. (Alabaster, AL). N-(7-nitrobenz-2-oxa-1,3-diazol-4-yl)-1,2-dihexadecanoyl-sn-glycero-3-phosphatidylethanolamine (NBD-PE, N360), p-xylene-bis-pyridinium bromide (DPX, X-1525) and 8-aminonaphthalene-1,3,6-trisulfonic acid, disodium salt (ANTS, A350) were purchased from Thermo Fisher Scientific (Waltham, MA).

### DNA constructs and site-directed mutagenesis

The details of all the constructs used are shown in Supp. Table 1. The pGEX4T-1 plasmids for expression of the various LC3/GABARAP proteins tagged with glutathione S-transferase (GST) (human LC3A, human LC3B, human LC3C, human GABARAP, human GABARAP and human GABARAPL2) were kindly provided by Dr. Ivanna Novak (School of Medicine, University of Split, Croatia). Note that, each of these LC3/GABARAP constructs was a truncated form lacking the C-terminal Gly. The Gly-exposed forms used in this work, such that no ATG4-mediated pre-processing was necessary, were constructed using a QuikChange site-directed mutagenesis kit (Stratagene, 200514). The sequences in all mutant constructs were confirmed by DNA sequencing analysis (Secugen, Madrid, Spain). The pGEX6P-1 plasmid for expression of human ATG3 was kindly provided by Dr. Isei Tanida (National Institute of Infectious Diseases, Tokyo, Japan).

### Recombinant protein expression and purification

LC3/GABARAP proteins and ATG3 were purified from soluble fractions of bacterial extracts obtained in the absence of detergents, and they were > 90% pure as evaluated by Coomassie Brilliant Blue-stained SDS-PAGE (Supp. Fig. 1a; 1b, line 2). *E. coli* BL21 (λDE3) cells were transformed with the appropriate plasmids. They were grown to A_600_ = 0.8 and protein expression was induced with 0.5 mM IPTG for 16 h at 20 °C. Following centrifugation at 4500 × *g* for 15 min, the pellet was resuspended and sonicated in breaking buffer (phosphate buffered saline (PBS) with protease-inhibitor mixture and 1 mM DTT). After removal of cellular debris by centrifugation at 30,000 × *g* for 30 min at 4 °C, the sample supernatant was incubated with 1 ml Glutathione Sepharose 4B (GE Healthcare, 17-0756-01) for 3 h at 4 °C to bind GST-tagged proteins. LC3/GABARAP proteins were cleaved with Thrombin Protease (GE Healthcare, 27–0846-01) overnight at room temperature in Thrombin Buffer (140 mM NaCl, 2.7 mM KCl, 10 mM Na_2_HPO_4_, 1.8 mM KH_2_PO_4_ (pH 7.3) with freshly added 1 mM DTT) and ATG3 protein was cleaved with PreScission Protease (GE Healthcare, 27-0843-01) for 4 h at 4 ºC in Buffer A (50 mM Tris–HCl pH 7.5, 150 mM NaCl, 1 mM EDTA with freshly added 1 mM DTT). After cleavage, they were eluted in Buffer A, then concentrated to 500 µl using Amicon Ultra-4 (4 mL, 3 kDa cutoff) (Millipore, UFC800324), and loaded onto a Superdex-75 10/300 GL size exclusion column (GE Healthcare, GE17-5174-01) equilibrated in Buffer A. Proteins were distributed in aliquots, flash-frozen and stored in 20% glycerol at − 80 °C until further use.

ATG12–ATG5-ATG16L1 (E3) was purified from soluble fractions of insect cell extracts obtained in the absence of detergents, and it was > 90% pure as evaluated by Coomassie Brilliant Blue-stained SDS-PAGE (Supp. Fig. 1b, line 3). For E3 expression, the Bac-to-Bac Baculovirus expression system was used. The pGEBdest vector containing a poli-cystronic construct (Supp. Table 1) with the ATG12 system necessary proteins for E3 formation [38] was transformed into DH10Bac *E. coli* cells. Blue/white colony selection was used to identify colonies containing the recombinant bacmid. The recombinant bacmid was isolated and 2.5 µg was used to transfect 10^6^ Sf9 insect cells using FuGENE transfection reagent (Promega, E2311). When the transfected cells demonstrated signs of late stage infection (typically around 72 h), the medium containing the free virus was harvested (V0) and used to produce a stock virus (V1) solution. V1 was used to further infect a 1-L culture of Sf9 cells at 0.8 − 1 × 10^6^/ml in SF921 medium containing penicillin–streptomycin. Cultures were harvested when cells reduced their viability to a maximum of 95–98%. They were pelleted down and further washed in PBS at 3315 × *g* for 10 min at 4 °C. Pellets were flash-frozen in liquid nitrogen and stored at − 80 °C until purification. Then, cell pellets were thawed and resuspended in ice cold buffer containing 50 mM Hepes, pH 7.5, 300 mM NaCl, 2 mM MgCl_2_, 1 mM DTT, complete protease inhibitors, Protease Inhibitor Cocktail, and Benzonase Nuclease. Cells were lysed on ice by extrusion in a tissue homogenizer, and lysates were cleared by centrifugation at 48,398 × *g* for 1 h at 4 °C. Supernatant was applied to a 5-ml StrepTactin column (GE Healthcare) to bind Strep-tagged proteins. Bound proteins were eluted with 2.5 mM desthiobiotin in 25 mM Hepes, pH 7.5, 150 mM NaCl, and 1 mM DTT. Fractions containing E3 were pooled, concentrated down to 500 µl using Amicon Ultra-15 (15 mL, 30 kDa cutoff), applied onto a Superose 6 column (Increase 10/ 300; GE Healthcare), and eluted in a buffer containing 25 mM Hepes, pH 7.5, 300 mM NaCl, and 1 mM DTT. Fractions containing pure E3 were pooled, distributed in aliquots, flash-frozen, and stored at − 80 °C until further use.

Mouse ATG7 (mATG7) was purified from soluble fractions of insect cell extracts obtained in the absence of detergents, and it was > 90% pure as evaluated by Coomassie Brilliant Blue-stained SDS-PAGE (Supp. Fig. 1b, line 1). It was also expressed in Sf9 insect cells and harvested following the same procedure described above for E3. See Supp. Table 1 for construct details. For purification, pellets were thawed and resuspended in ice cold buffer containing 50 mM Hepes, pH 7.5, 300 mM NaCl, 10 mM imidazole, 2 mM MgCl_2_, 2 mM β-mercaptoethanol, complete protease inhibitors (Roche), Protease Inhibitor Cocktail (Sigma), and Benzonase Nuclease (Sigma). Cells were lysed on ice by extrusion in a tissue homogenizer, and lysates were cleared by centrifugation at 48,398 × *g* for 1 h at 4 °C. Supernatant was applied to a 5-ml nickel-nitrilotriacetic acid (Ni–NTA) column (GE Healthcare) and eluted via a stepwise imidazole gradient (50, 75, 100, 150, 200, and 300 mM). Protein eluted in fractions containing 150 mM imidazole. These fractions were pooled, concentrated, applied onto a Superdex 200 10/300 GL (GE Healthcare), and eluted in a buffer containing 25 mM Hepes, pH 7.5, 150 mM NaCl, and 1 mM DTT. Fractions containing pure mATG7 were pooled, concentrated, flash-frozen in liquid nitrogen, and stored at − 80 °C.

### Liposome preparation

The appropriate lipids (ePC/DOPE/PI/DOG, 33:55:10:2 mol ratio) were mixed in organic solution, and the solvent was evaporated to dryness under a N_2_ stream. Then, the sample was kept under vacuum for 1 h to remove solvent traces. The lipids were swollen in System Buffer (150 mM NaCl, 50 mM Tris, pH 7.5) in order to obtain multilamellar vesicles (MLVs). Large unilamellar vesicles (LUV) were produced from MLV according to the extrusion method described by Mayer et al. [40]. They were subjected to 10 freeze/thaw cycles and then extruded through a LIPEX Liposome Extrusion System (Transferra Nanosciences, Burnaby, CA) using 0.05-μm pore size Nuclepore filters (Whatman, 110605). Vesicle size was checked by quasi-elastic light scattering using a Malvern Zeta-Sizer 4 spectrometer (Malvern Instruments, Malvern, UK). LUV had an average diameter of ≈ 80 nm. Phospholipid concentration was determined by phosphate analysis [41].

### In vitro enzymatic lipidation reaction

Purified ATG7 (0.5 µM), ATG3 (1 µM), MgCl_2_ (1 mM), E3 (0.1 µM) (when indicated) and the pertinent member of the LC3/GABARAP family member with an exposed Gly C-terminal (5 µM) were mixed with liposomes (0.4 mM total lipid) in System Buffer (50 mM Tris pH 7.5, 150 mM NaCl) to a final volume of 100 µl (see Fig. 2 and Supp. Fig. 2 legends for details). Reactions were performed at 37 °C and initiated by the addition of ATP (5 mM). 15 μl of the reaction mixture was sampled at each time point (0, 5, 10, 15, 30 and 60 min), mixed with 3 μl of 6 × Protein Loading dye and heated at 60 °C for 10 min to stop the reaction. Lipidation was analyzed in SDS-PAGE gels by using Comassie Brilliant Blue staining. Lipidation reactions performed in Supp. Fig. 2 were also analyzed using a VersaDoc MP 4000 Imaging System to detect NBD fluorescence. The gels of three independent experiments were quantified using ImageJ. The amounts of LC3/GABARAP and LC3C/GABARAP–PE at each time point were measured as the area below the corresponding absorption peak. The percent LC3/GABARAP–PE relative to total protein (% lipidation) was calculated at each time point and plotted as a function of time.

### Vesicle flotation assay

Protein interaction with membranes was assessed using flotation in sucrose gradients. All the liposome and protein concentrations used were increased (by fivefold) with respect to the other assays, all proportions being otherwise kept, to allow detection of E3 in the gels (see Fig. 3 and Supp. Fig. 3 legends for details). Liposomes were incubated with the indicated proteins for 30 min at 37 °C in System Buffer. The protein/lipid mix was adjusted to 1.4 M sucrose concentration in 300 μl and transferred to a centrifuge tube. This first (bottom) layer was overlaid with successive solutions containing 0.8 M (400 μl) and 0.5 M (300 μl) sucrose. The three-layer gradients were centrifuged in a TLA-120.2 rotor (Beckman Coulter, Brea, CA, US) at 355,040 × g for 50 min at 4 °C. After centrifugation, four 250-µl fractions were collected, starting from the bottom. Proteins were detected in SDS-PAGE gels using Comassie Brilliant Blue staining. Densitometry of the protein bands was performed using ImageJ software, and the percent liposome-bound protein was estimated from the band intensities measured in the third + fourth fractions (floating vesicle fractions), relative to the total sum of intensities measured in all fractions.

### Tethering assays

Liposome tethering/aggregation was monitored in a Varian Cary 300 (Agilent Technologies, Santa Clara, CA) spectrophotometer as an increase in turbidity (absorbance at 400 nm) of the sample (see also Supp. Fig. 4a). All assays were carried out at 37 °C with continuous stirring [35]. See Fig. 4 legend for protein and lipid concentration details.

### Total and inner lipid mixing assay

A fluorescence resonance energy transfer (FRET) assay was used to monitor inter-vesicular membrane lipid mixing (see also Supp. Fig. 4b). [42]. The appropriate LUV containing 1.5 mol % NBD-PE and 1.5 mol % Rho-PE (labeled in the head group) were mixed with a ninefold excess of unlabeled LUV (see Fig. 5 and Fig. 6a, b legend for protein and lipid concentration details). NBD-PE emission was monitored in a Fluorolog^®^-3 (Horiba Jobin Yvon, Edison, NJ) spectrofluorometer with constant stirring at 37 °C. NBD emission was monitored at 530 nm with the excitation wavelength set at 465 nm (slits at 4 nm). A 515 nm cutoff filter was placed between the sample and the emission monochromator to avoid scattering interference. Inner monolayer lipid mixing was measured using asymmetrically labeled membrane vesicles produced by the quenching of the outer leaflet NBD-PE fluorescence upon addition of sodium dithionite [43]. Excess dithionite was removed by gel filtration in Sephadex G-25 M, using System Buffer for elution. 100% inter-vesicular membrane lipid mixing and 100% inner-monolayer lipid mixing were established by adding 10 µL of 10% (v/v) Triton X-100. The extent of lipid mixing was quantified on a percentage basis according to the equation: (F_t_-F_0_/F_100_-F_0_) × 100, where F_t_ is the measured NBD fluorescence of protein-treated LUV at time t, F_0_ is the initial NBD fluorescence of the LUV suspension before ATP addition, and F_100_ is the NBD fluorescence value after complete disruption of LUV by addition of Triton X-100. Details for the inter-vesicular lipid mixing assay can be found in Goñi et al. [44].

### Vesicle contents leakage assay

Leakage of vesicle contents was monitored by the ANTS/DPX leakage assay [45]. Liposomes were swollen in ANTS/DPX buffer (20 mM ANTS, 70 mM DPX, 50 mM Tris, 40 mM NaCl, pH 7.5). Non-encapsulated ANTS and DPX were removed by gel filtration in Sephadex G-25 M, using System Buffer for elution (see Supp. Fig. 10 legend for protein and lipid concentration details). ANTS emission was monitored at 520 nm with the excitation wavelength set at 355 nm (slits at 4 nm). To establish the 100% leakage signal, 10 µL of 10% (v/v) Triton X-100 was added. The extent of leakage was quantified on a percentage basis according to the equation: (*F*_*t *_− *F*_0_/*F*_100 _− *F*_0_) × 100, where *F*_*t*_ is the measured ANTS fluorescence of protein-treated LUV at time *t*, *F*_0_ is the initial ANTS fluorescence of the LUV suspension before ATP addition, and *F*_100_ is the ANTS fluorescence value after complete disruption of LUV by addition of Triton X-100. Details for the vesicle contents leakage assay can be found in [44].

### Aqueous contents mixing assay

Inter-vesicular aqueous contents mixing was monitored by the ANTS/DPX mixing assay [45]. Three types of liposomes were prepared. Liposomes were swollen in either ANTS buffer (39 mM ANTS, 50 mM Tris, 72 mM NaCl, pH 7.5), in DPX buffer (140 mM DPX, 50 mM Tris, 10 mM NaCl, pH 7.5), or in ANTS/DPX buffer (20 mM ANTS, 70 mM DPX, 50 mM Tris, 40 mM NaCl, pH 7.5). Non-encapsulated ANTS and/or DPX were removed by gel filtration in Sephadex G-25 M, using System Buffer for elution. All buffers had the same osmolarity (see Fig. 6c, d and Supp. Fig. 11 legend for protein and lipid concentration details). ANTS emission was monitored at 520 nm with the excitation wavelength set at 355 nm (slits, 1 nm). 0% vesicle contents mixing was set by using a 1:1 mixture of ANTS- and DPX-containing liposomes. 100% contents mixing corresponded to the fluorescence of the vesicles containing co-encapsulated ANTS and DPX. The extent of aqueous contents mixing was quantified on a percentage basis according to the equation: (− (*F*_*t* _− *F*_0_/*F*_100 _− *F*_0_)) × 100, where F_t_ is the measured ANTS fluorescence of protein-treated LUV at time t, F_0_ is the initial ANTS fluorescence of the LUV suspension before protein addition, and F_100_ is the ANTS fluorescence value of the vesicles containing co-encapsulated ANTS/DPX. Details for the aqueous contents mixing assay can be found in [44].

### Cryo-EM sample preparation and image collection

Conjugation reactions (see Fig. 7 legend for protein and lipid concentration details) were performed at 37 °C for 90 min with continuous stirring and the reaction mixtures loaded on freshly glow-discharged 300-mesh R2/2 Quantifoil holey carbon grids (Quantifoil Micro Tools GmbH). Vitrification was performed on a LEICA GP2 automatic plunge freezer (LEICA microsystems) maintained at 8 °C at a relative humidity close to saturation (90% rH). Grids were loaded with 4 µL sample solutions for 30 s, blotted with absorbent standard filter paper, and plunged into a liquid ethane bath. The vitrified grids were removed from the plunger and stored under liquid nitrogen.

Imaging of cryo-EM samples was performed on a JEM-2200FS/CR (JEOL Europe, CIC bioGUNE, Spain) transmission electron microscope operated at 200 kV and images were recorded under low-dose conditions, with a total dose of the order of 30–40 electrons/Å^2^ per exposure, at defocus values ranging from − 1.5 to − 4.0 µm. The in-column Omega energy filter of the microscope helps to record images with improved signal-to-noise ratio (SNR) by zero-loss filtering, using an energy selecting slit width of 20 eV centered at the zero-loss peak of the energy spectra. Digital images were recorded on a GATAN K2 summit direct detection camera 4 K × 4 K (5 µm pixels) (Gatan Inc., Pleasanton, CA) using Digital Micrograph (Gatan Inc.) software, at a nominal magnification of 30,000 × , resulting in final sampling of 1.3 Å/pixel.

## Results

### LC3/GABARAP protein in vitro lipidation in the presence of E3

**Fig. 1:**
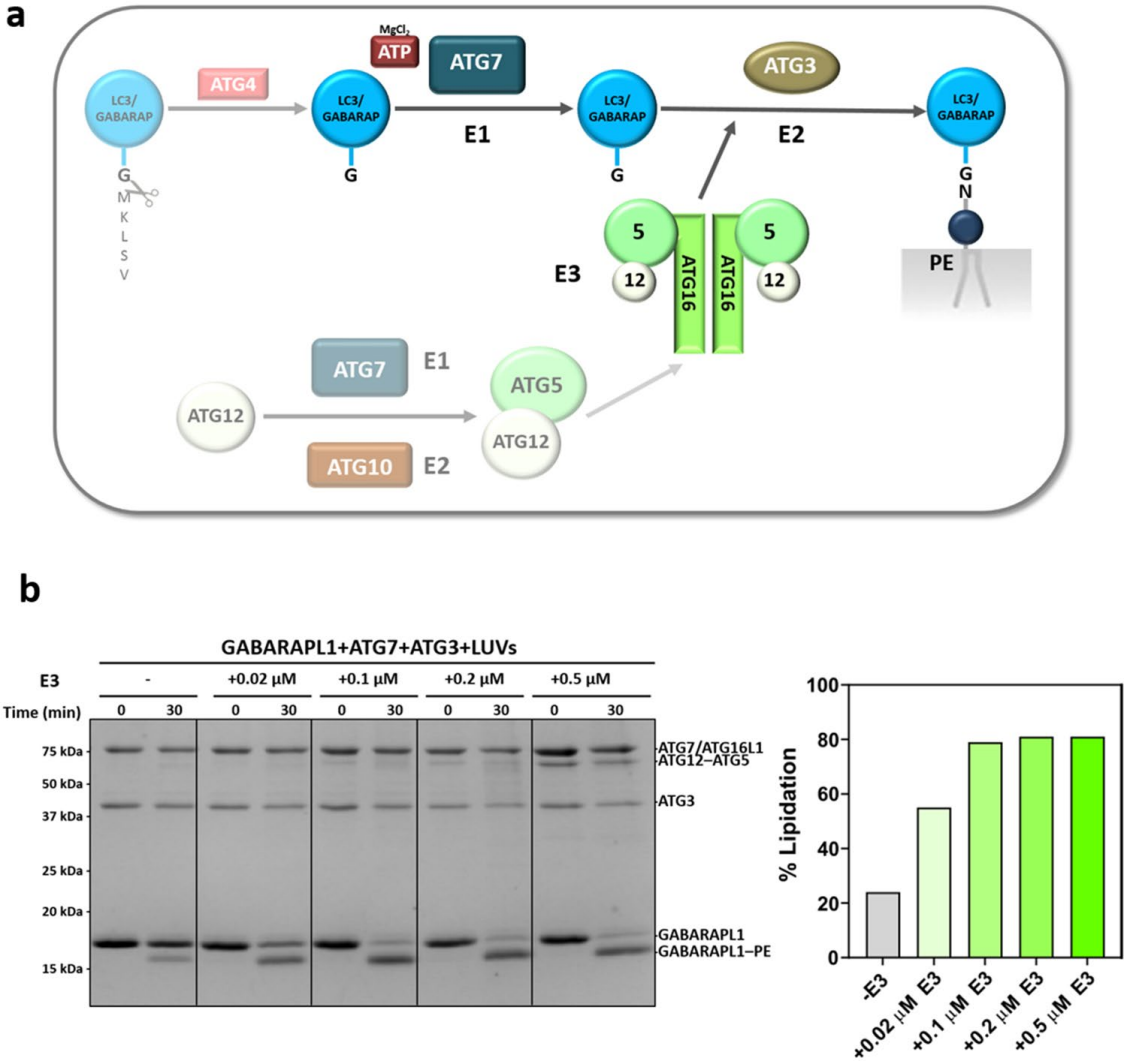
LC3/GABARAP in vitro lipidation in the presence of E3. **a** Schematic representation of the reconstituted LC3/GABARAP lipidation system used in this study. ATP promoted ATG7 (E1-like), ATG3 (E2-like) and ATG12-ATG5-ATG16 (E3-like) actions triggering LC3/GABARAP conjugation to PE in PE-containing liposomes. LC3/GABARAP proteins had their Gly C-terminal exposed to avoid the requirement of ATG4 participation. E3 was expressed in insect cells. **b** In vitro GABARAPL1 lipidation assay in the presence of increasing E3 concentrations. Left: 0.5 µM ATG7, 1 µM ATG3, and 5 µM GABARAPL1 (see Supp. Fig. 1b for further details) were mixed with 0.4 mM LUV (ePC:DOPE:PI:DOG (33:55:10:2 mol ratio)), in the absence (-) or in the presence of different E3 concentrations (0.02, 0.1, 0.2, 0.5 μM), and incubated at 37 °C in System Buffer containing MgCl_2_ and ATP. Aliquots were retrieved 0 and 30 min after ATP addition and loaded on a 15% SDS–polyacrylamide gel. Right: Percent lipidated protein, quantified as described under Methods

Conjugation of LC3/GABARAP proteins to PE in the phagophore membranes is an ATP-dependent process requiring the concerted action of the LC3/GABARAP and ATG12 conjugation systems. For simplicity, the LC3/GABARAP proteins used in this study (Supp. Fig. 1a) were modified in order to expose the C-terminal Gly, thus avoiding the ATG4 step, and E3 was produced in insect cells by the co-expression of its three subunits together with ATG7 and ATG10, as described in Fracchiolla et al. [38] (Fig. 1a). Moreover, vesicles were prepared with a composition [27] that would ensure a basal degree of protein lipidation even in the absence of E3. This experimental approach should allow us to assess the effect of E3 on the ability of the different LC3/GABARAP proteins to produce membrane tethering or fusion.

To determine the E3 concentration that was required under the above conditions, the lipidation level of one of the proteins, GABARAPL1, was assessed 30 min after ATP addition using different E3 concentrations. 0.1 μM E3 (corresponding to a 1:50 E3:GABARAPL1 mol ratio) was enough for a ≈ 80% GABARAPL1 lipidation. Adding more complex, up to 0.5 µM, did not increase lipidation under the indicated experimental conditions (Fig. 1b). These results showed that, when a high proportion of PE was present, lipidated protein could be obtained even in the absence of E3, and that low levels of E3 were enough for achieving almost full lipidation.

### E3 increases and accelerates LC3/GABARAP lipidation

**Fig. 2:**
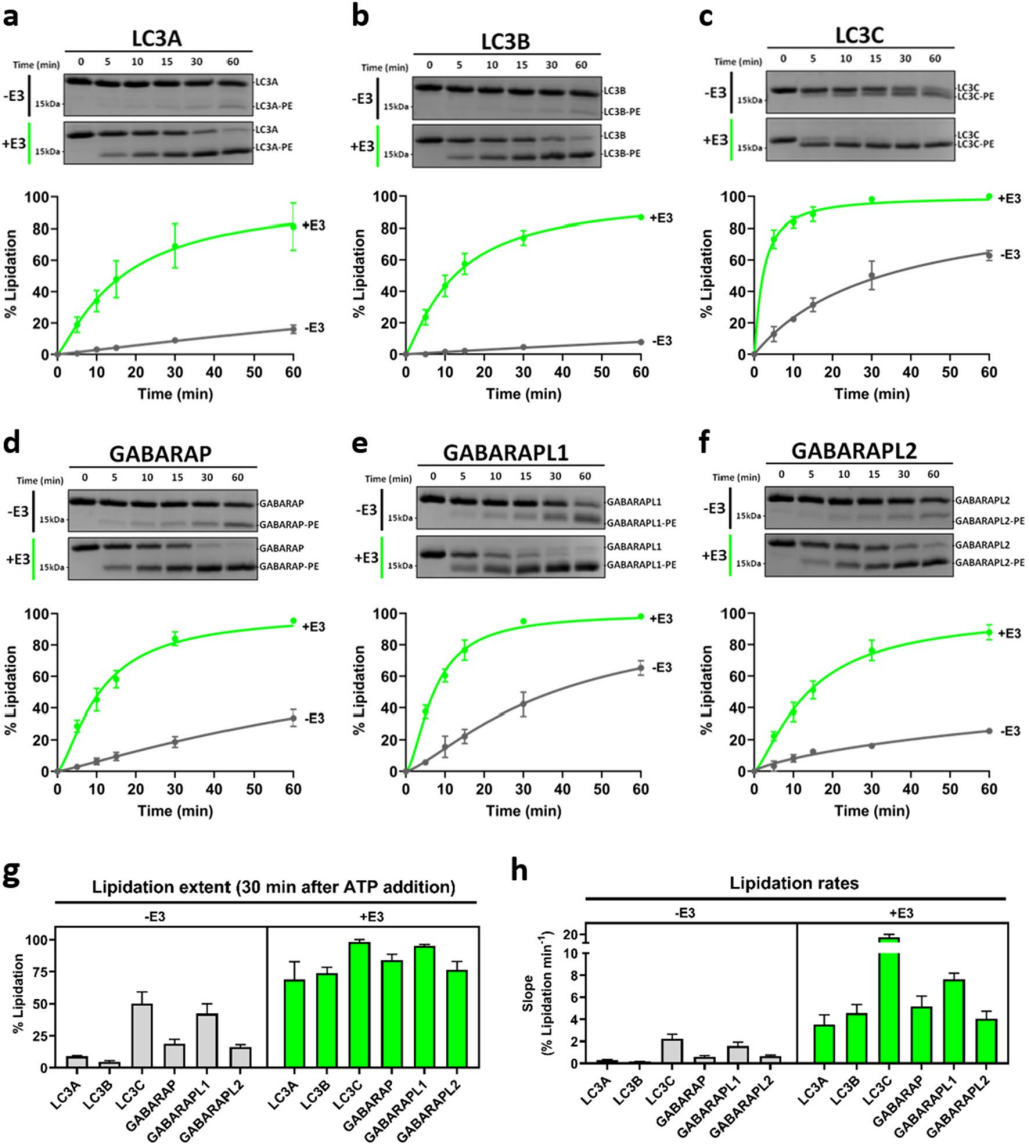
E3 increases and accelerates LC3/GABARAP lipidation. **a–f** In vitro LC3/GABARAP lipidation assay: 0.5 µM ATG7, 1 µM ATG3, and 5 µM of the indicated LC3/GABARAP protein were mixed with 0.4 mM LUV (ePC:DOPE:PI:DOG (33:55:10:2 mol ratio)), in the absence (− E3, gray) or presence (+ E3, green) of 0.1 µM E3 and incubated at 37 °C. After ATP addition, aliquots retrieved at pre-fixed time points were loaded on a 15% SDS–polyacrylamide gel. Upper panel: Crop of representative lipidation gels corresponding to the LC3/GABARAP protein region (An example of a full gel can be seen in Fig. 1b). Lower panel: Time-course of the protein percent lipidation. **g** Percent lipidated LC3/GABARAP 30 min after ATP addition in the absence (left) or presence (right) of E3.** h** Initial lipidation rates of the various LC3/GABARAP in the absence (left) or presence (right) of E3. Data are means ± SD (*n = *3)

For a quantitative study of E3 effect on lipidation, the various LC3/GABARAP proteins were added to a mix of ATG7, ATG3, PE-containing LUV and, when indicated, E3. Aliquots were collected at pre-fixed times after ATP addition (0, 5, 10, 15, 30 and 60 min) (Fig. 2a–f), and the lipidated and non-lipidated forms were resolved by SDS-PAGE. Lipidation caused a faster migration of the proteins, as confirmed by the appearance of a fluorescent faster migrating band when liposomes containing NBDtail-PE were used (Supp. Fig. 2).

When comparing the results in the absence or presence of E3, a clear E3-dependent increase in the lipidation rates and extents was observed for all the LC3/GABARAP proteins (Fig. 2a–f). In the absence of E3, the extents of lipidation after 30 min (Fig. 2g, − E3 panel) were highest for LC3C and GABARAPL1 (> 30%), followed by GABARAP, GABARAPL2 (> 10%), LC3A and LC3B (> 5%). When E3 was present (Fig. 2g, + E3 panel), all the proteins were > 50% lipidated, with small differences between the various homologs. When comparing lipidation rates (Fig. 2h), LC3C and GABARAPL1 exhibited the fastest lipidation in the absence of E3 (Fig. 2h, − E3 panel). When E3 was present, all reactions went faster and LC3C exhibited the highest rate, up to 15% lipidated protein/min, followed by GABARAPL1 (Fig. 2h, + E3 panel). These results showed differences in the lipidation capacity of the LC3/GABARAP family in the absence of E3 and confirmed the ability of E3 to increase and accelerate LC3/GABARAP protein lipidation. The E3 effect was particularly visible in LC3A and LC3B lipidation, since lipidation of those proteins in the absence of the complex was very low.

Furthermore, protein-liposome interaction during GABARAPL1 lipidation was analyzed using a flotation assay. Even before adding ATP (Supp. Fig. 3, -ATP), an initial interaction of the lipidation machinery with membranes was seen. All the E3 appeared in the bound fraction together with part of ATG3 and ATG7, and a small percentage of GABARAPL1. Upon ATP addition, an increased interaction of all the proteins was observed. As expected, the lipidated band of GABARAPL1 appeared in the bound fraction, together with ATG3, E3 and part of ATG7 (Supp. Fig. 3, + ATP). This suggests that ATP addition did not only allow lipidation, but it also enhanced ATG7 and ATG3 binding to vesicles.

### In the presence of ATG3, low concentrations of E3 allow vesicle tethering.

**Fig. 3:**
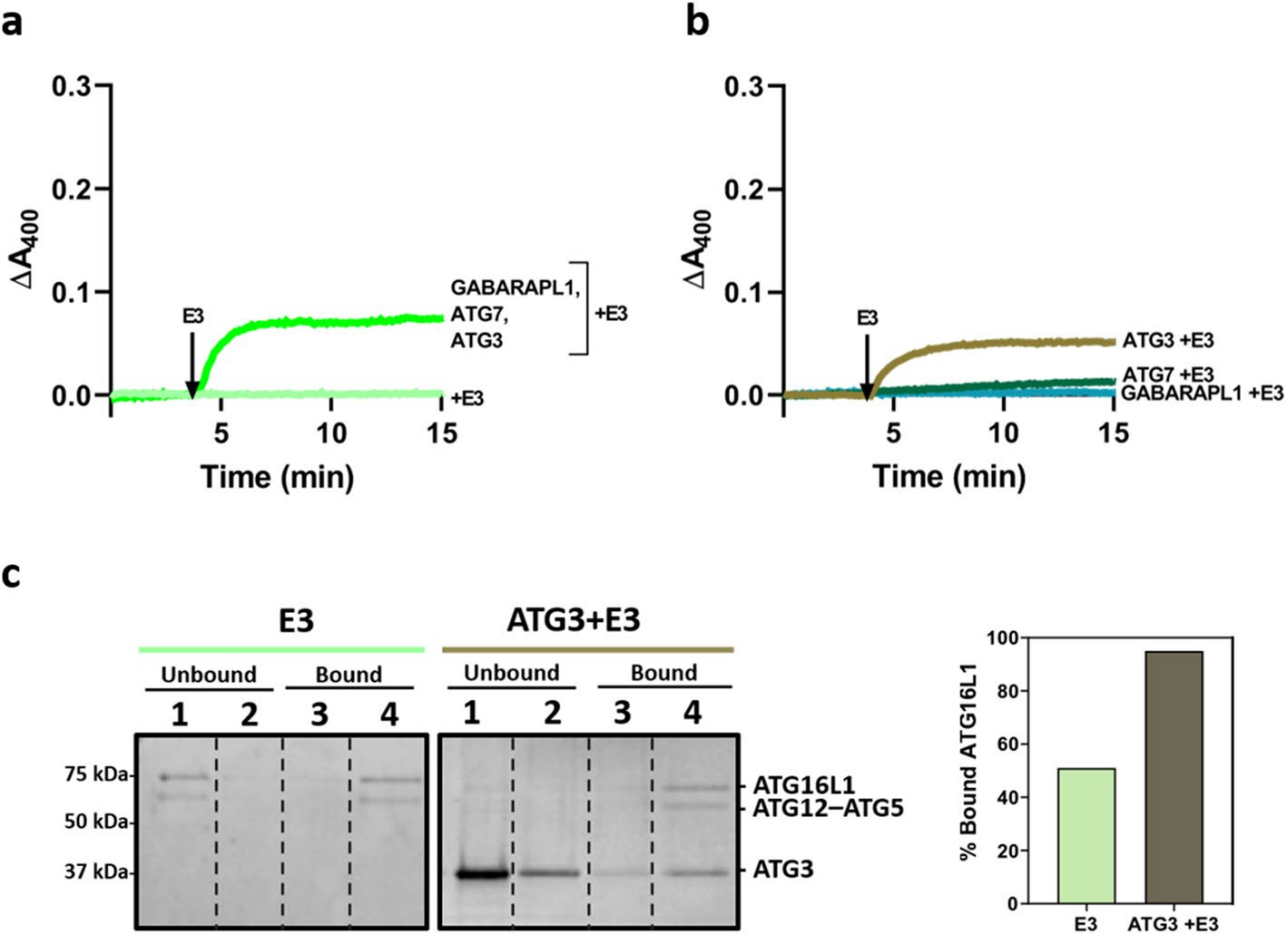
In the presence of ATG3, low concentrations of E3 allow vesicle tethering. **a**, **b** Changes in turbidity (ΔA_400_), as a signal of vesicle tethering, were measured after E3 addition. **a** Tethering of 0.4 mM LUV [ePC:DOPE:PI:DOG (33:55:10:2 mol ratio)] caused by 0.1 µM E3 alone (light green line) or in the presence of 5 µM GABARAPL1, 0.5 µM ATG7 and 1 µM ATG3 (green line). **b** Tethering of 0.4 mM LUV [ePC:DOPE:PI:DOG (33:55:10:2 mol ratio)] caused by addition of 0.1 µM E3 in the presence of 5 µM GABARAPL1 (blue line), 0.5 µM ATG7 (dark green line) or 1 µM ATG3 (ochre line). **c** Interaction of E3 with membranes in the absence and in the presence of ATG3 measured by a vesicle flotation assay. Protein and liposome concentrations were increased by fivefold to allow detection of E3 in the gels. 0.5 µM E3 was incubated with 2 mM LUV [ePC:DOPE:PI:DOG (33:55:10:2 mol ratio)] in the absence or presence of 2.5 µM ATG3. Left: SDS-PAGE/Coomassie Brilliant Blue-stained gels of the fractions obtained from E3 vesicle flotation assays in the absence (-ATG3 panel) or presence of ATG3 (+ ATG3 panel). Protein found in fractions 3 + 4 was taken as bound protein. Right: Percent ATG16L1 bound to liposomes in the absence or presence of ATG3, quantified by gel densitometry

The tethering ability of E3 had been previously described in yeast [11]. In the present investigation, the capacity of low concentrations (0.1 µM) of human E3 to cause vesicle tethering was tested. Liposome tethering/aggregation is usually assessed as an increase in suspension turbidity (Supp. Fig. 4a). When E3 alone was added to liposomes, no change in turbidity (ΔA_400_) was detected (Fig. 3a, light green line). However, when added to a mixture composed of liposomes, GABARAPL1, ATG7 and ATG3, a fast increase in A_400_ was observed. Vesicle tethering started as soon as E3 was added and reached a plateau in about 5 min (Fig. 3a, green line). The role of the various components in the observed tethering effect was dissected next.

When, in addition to liposomes, only GABARAPL1 was present, E3 addition did not cause any increase in turbidity (Fig. 3b, blue line). Thus, the E3 tethering effect would require either ATG3 or ATG7, or a combination of both. Experiments performed with each of them separately showed that ATG3 was the main agent co-operating with E3 in the tethering effect (Fig. 3b). This effect was E3 concentration dependent (Supp. Fig. 5). To further understand why E3 was able to promote vesicle tethering when ATG3 was present, a liposome flotation assay was performed comparing the E3 ability to interact with membranes in the absence or presence of ATG3. All the E3 was vesicle-bound when ATG3 was present (Fig. 3c). Thus, ATG3 enhanced E3 interaction with the membrane, allowing an initial stage of liposome tethering.

### LC3/GABARAP-promoted vesicle tethering is enhanced and accelerated by the E3-induced increase in lipidation

**Fig. 4:**
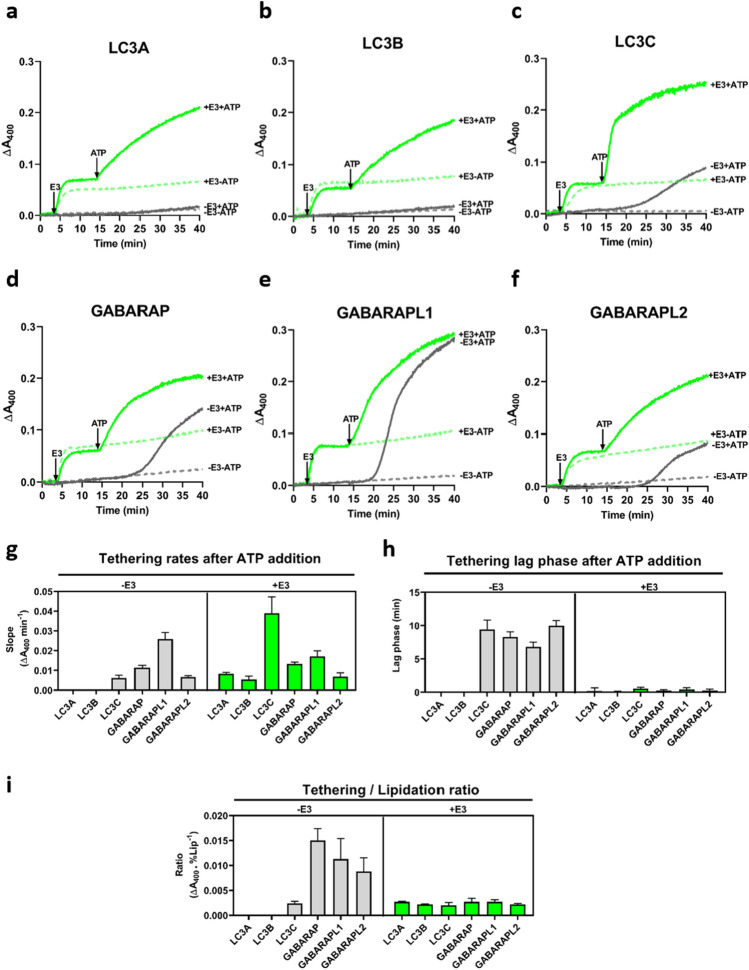
E3 effect on lipidation enhances and accelerates LC3/GABARAP-promoted vesicle tethering. Membrane tethering activities by lipidated LC3/GABARAP proteins in the absence and presence of E3. 0.4 mM LUV [ePC:DOPE:PI:DOG (33:55:10:2 mol ratio)], 0.5 µM ATG7, 1 µM ATG3, and 5 µM of the pertinent LC3/GABARAP family member were mixed. After 4 min, either 0.1 µM E3 (+ E3, green lines) or buffer (− E3, gray lines) was added, and 10 min later, ATP (+ ATP, solid lines) or buffer (-ATP, dashed lines) was added. Changes in absorbance at 400 nm (ΔA_400_), as an indication of vesicle tethering, were measured. **a–f** Representative curves of the indicated LC3/GABARAP member in the four conditions analyzed: − E3 -ATP (gray dashed lines), − E3 + ATP (gray solid lines), + E3 -ATP (green dashed lines), + E3 + ATP (green solid lines). **g** Tethering rates after ATP addition in the absence (left) or in the presence (right) of E3. LC3A or LC3B did not cause any measurable activity. Data are means ± SD (*n = *3). **h** Lag phase of tethering activity after ATP addition in the absence (left) or in the presence (right) of E3. Data are means ± SD (*n = *3). **i** Tethering/lipidation ratios: Final tethering levels caused by lipidated LC3/GABARAP proteins related to the percent lipidated protein present, in the absence (left) or in the presence (right) of E3. See also Supp. Fig. 9. Data are means ± SD (*n = *3)

The capacity of the different lipidated LC3/GABARAP to induce vesicle tethering was comparatively tested (Fig. 4a–f, Supp. Fig. 4a). To this aim, PE-containing LUV, ATG3, ATG7, and the pertinent LC3/GABARAP family member were mixed. After 4 min, either E3 (+ E3, green lines) or buffer (− E3, gray lines) was added, and 10 min later, ATP (+ ATP, solid lines) or buffer (-ATP, dashed lines) was equally added. In addition, lipidation was assessed at the end of the experiment (25 min after ATP addition) (Supp. Fig. 6, Tethering panels).

When proteins could not be lipidated (in the absence of ATP) and E3 was not present (Fig. 4a–f, − E3-ATP, gray dashed lines), no change in turbidity (A_400_) was observed. However, as described in the previous section for GABARAPL1 (Fig. 3a), E3 addition caused an initial tethering activity for all LC3/GABARAP proteins (Fig. 4a–f, + E3, green lines).

After ATP addition, so that proteins could be lipidated, tethering was observed in almost all cases (Fig. 4a–f, solid lines). In the absence of E3 (Fig. 4a–f, E3, + ATP, gray solid lines), the protein eliciting the fastest and most extensive tethering was GABARAPL1, followed by GABARAP, GABARAPL2 and LC3C. LC3A and LC3B had no measurable effect (Fig. 4a–f, − E3 panel). However, if E3 was present (Fig. 4a–f, + E3, + ATP, green solid lines), meaning that lipidation was faster and higher, all the proteins, includingLC3A and LC3B, were able to induce some tethering, LC3C achieving by far the fastest rates (Fig. 4g, + E3 panel). Moreover, once LC3C or GABARAPL1 was fully lipidated (1 h after ATP addition), further increases in E3 concentration failed to cause any additional tethering (Supp. Fig. 7). These results suggest that the higher tethering levels observed after ATP addition in the presence of E3 were the result of E3 effect on lipidation and not a direct effect of E3 on tethering.

All four LC3/GABARAP proteins that induced a measurable extent of tethering in the absence of E3 (LC3C, GABARAP, GABARAPL1, and GABARAPL2) also showed a considerable lag phase (Fig. 4h). A negative correlation appeared to exist between rate (maximum slope) and lag time (Fig. 4g, h, − E3 panel). However, when E3 was present, no lag phase was detected, and vesicle tethering started immediately after adding ATP (Fig. 4h). This could indicate that a minimum degree of lipidation, achieved faster when E3 was present, would be required for tethering to start.

There was in general a good parallelism between the time courses of LC3/GABARAP protein lipidation and LC3/GABARAP-induced vesicle tethering (Supp. Fig. 8). However, some peculiarities should be considered. (a) When E3 was not present, even if LC3C lipidation level was similar to that of GABARAPL1, both the extent and rate of vesicle tethering were lower (Supp. Fig. 8a, b, gray lines). (b) LC3A and LC3B, in the absence of E3, did not induce vesicle tethering, probably because of the low lipidation level (< 10%) achieved. However, GABARAP or GABARAPL2, with a slightly higher lipidation level, was able to cause a markedly higher extent of liposome tethering (Supp. Fig. 9a, b, gray bars). (c) E3 increased lipidation levels in all cases, however, at variance with the LC3 subfamily, the extent of tethering was similar with and without E3 for GABARAP and GABARAPL2 and lower in the presence of E3 for GABARAPL1 (Supp. Fig. 9a, b).

When the ratio between the extent of vesicle tethering and the percent lipidated protein at a given time (25 min after ATP addition was chosen for convenience) was computed (Fig. 4i), a clear difference between both subfamilies was observed in the absence of E3 (Fig. 4i, − E3 panel). However, in the presence of E3, when all homologs were lipidated by ≥ 50%, and their ability to induce vesicle tethering was quite similar (Supp. Fig. 9, green bars), the tethering/lipidation ratio was also similar for all proteins (Fig. 4i, + E3 panel). This could suggest a different lipidation threshold for each LC3/GABARAP family member, above which each of them would be able to induce vesicle tethering. This lipidation threshold would be lower for the GABARAP subfamily.

### E3 hampers LC3/GABARAP protein capacity to induce inter-vesicular lipid mixing

**Fig. 5:**
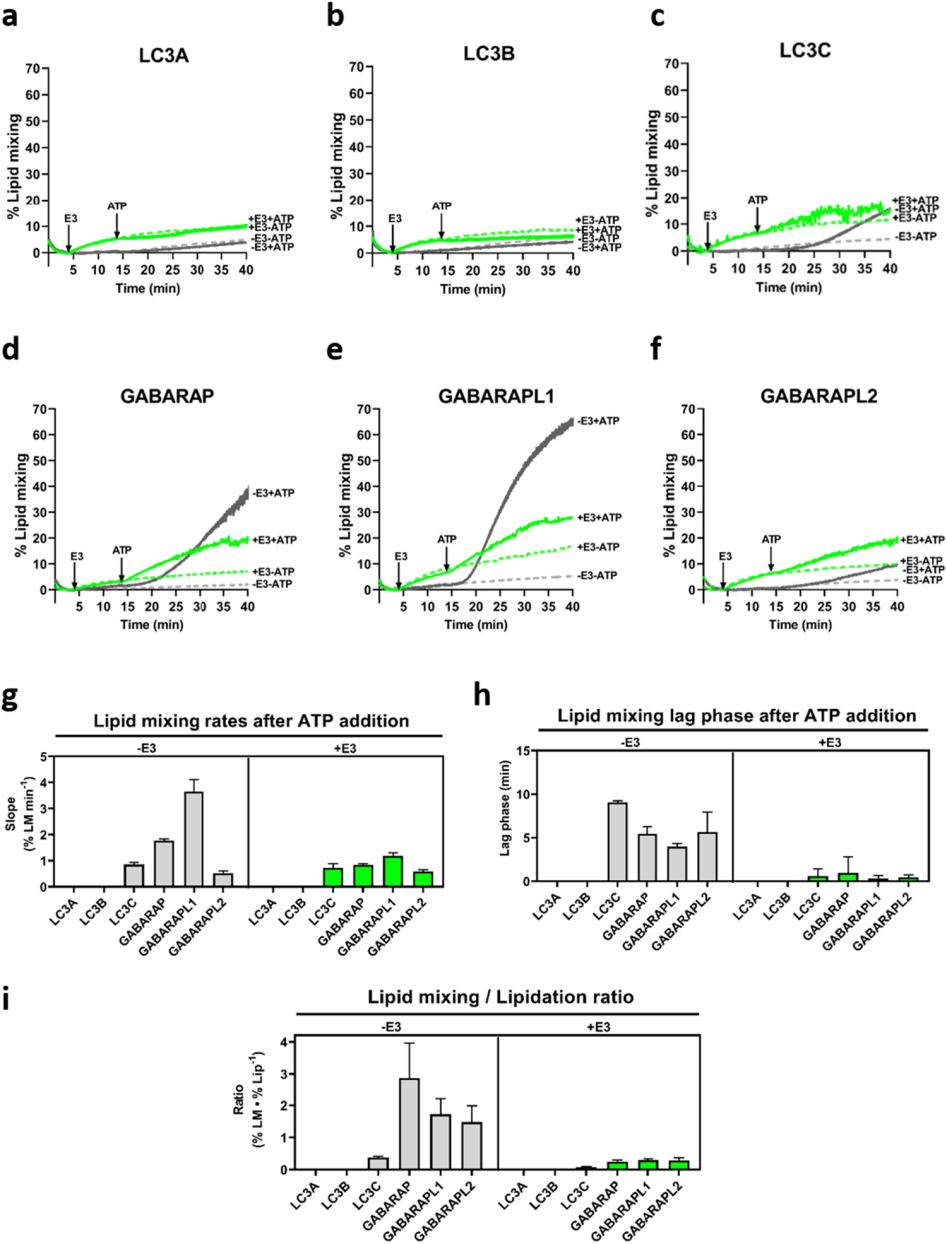
E3 hampers LC3/GABARAP capacity to induce inter-vesicular lipid mixing. Membrane lipid mixing activities by lipidated LC3/GABARAP proteins in the absence and in the presence of E3 were monitored with the NBD-PE/Rho-PE lipid dilution assay. 0.4 mM unlabeled and (NBD-PE + Rho-PE)-labeled liposomes (9:1) were mixed with 0.5 µM ATG7, 1 µM ATG3, and 5 µM of the pertinent LC3/GABARAP family member. After 4 min, either 0.1 µM E3 (+ E3, green lines) or buffer (− E3, gray lines) was added, followed 10 min later by ATP (+ ATP, solid lines) or buffer (-ATP, dashed lines). Increases in NBD fluorescence detection, as a signal of lipid mixing of labeled and unlabeled vesicles, were measured and the percentage of lipid mixing was calculated. See Methods for details. **a–f** Representative curves of the indicated LC3/GABARAP member in the four conditions analyzed: − E3 -ATP (gray dashed lines), − E3 + ATP (gray solid lines), + E3 -ATP (green dashed lines), + E3 + ATP (green solid lines). **g** Lipid mixing rates after ATP addition in the absence (left) or in the presence (right) of E3. LC3A or LC3B did not cause any measurable activity. Data are means ± SD (*n = *3). **h** Lag phase of lipid mixing after ATP addition in the absence (left) or in the presence (right) of E3. Data are means ± SD (*n = *3). **i** Final lipid mixing levels caused by lipidated LC3/GABARAP proteins related to the percent lipidated protein present, in the absence (left) or in the presence (right) of E3. Data are means ± SD (*n = *3)

Previous studies [15, 33, 35] had shown that at least some of the LC3/GABARAP proteins were able to induce inter-vesicular lipid mixing. The present study has found that the lipidated LC3/GABARAP proteins were able to induce vesicle tethering, and that this process was enhanced and accelerated by E3. A further step in our study consisted of checking the LC3/GABARAP protein ability to induce inter-vesicular lipid mixing and liposome fusion (Supp. Fig. 4b), and analyzing how E3 affected the process. First, we examined whether the small extent of tethering caused by E3 addition to the lipidation machinery also caused lipid mixing. In fact, a small lipid mixing effect was observed prior to ATP addition in all cases (Fig. 5a–f, + E3, green lines).

With the whole set of proteins, except E3, the results were in agreement with the lipidation and vesicle tethering observations (Fig. 5a–f and Supp. Fig. 8, gray solid lines). Lipidation levels were as in the tethering assays (Supp. Fig. 6). LC3A and LC3B were not able to induce lipid mixing. GABARAPL1 was the fastest and most effective inducer of inter-vesicular lipid mixing, followed by GABARAP (Fig. 5g, − E3 panel). Although LC3C lipidation levels were similar to those of GABARAPL1 (Supp. Fig. 9a, gray bars), its effect on lipid mixing was low, and similar to that of GABARAPL2 (Supp. Fig. 9c, gray bars). The four LC3/GABARAP proteins that induced a measurable extent of lipid mixing (LC3C, GABARAP, GABARAPL1, GABARAPL2) showed a lag phase before activity started, pointing again to a required threshold of protein lipidation before lipid mixing became detectable (Fig. 5h, − E3 panel). Moreover, the ‘lipid mixing/lipidation ratio’ revealed a clear difference between the two subfamilies (Fig. 5i, − E3 panel), as previously observed for vesicle tethering (Fig. 4i, − E3 panel). This could indicate, again, that the lipidation threshold would be lower for the GABARAP subfamily members.

However, at variance with the lipidation and tethering observations, E3 effect on lipidation (Supp. Fig. 8) did not increase the LC3/GABARAP protein capacity to promote lipid mixing (Fig. 5a–f and g). As seen in Supp. Fig. 8 and Supp. Fig. 9, in the case of LC3A and LC3B, the lipidated protein was able to cause vesicle tethering in the presence of E3, but it did not induce lipid mixing. For LC3C, the fast and extensive tethering observed in presence of E3 did not imply a comparable degree of lipid mixing. GABARAPL2 exhibited a similar behavior in the presence and absence of E3. For GABARAP and GABARAPL1, both the extents and rates of lipid mixing were decreased in the presence of E3. These results indicate that the presence of E3, which enhanced protein lipidation and vesicle tethering, reduced, by contrast, their lipid mixing ability.

### GABARAP and GABARAPL1 cause membrane hemifusion but are poor inducers of vesicle-vesicle fusion

**Fig. 6:**
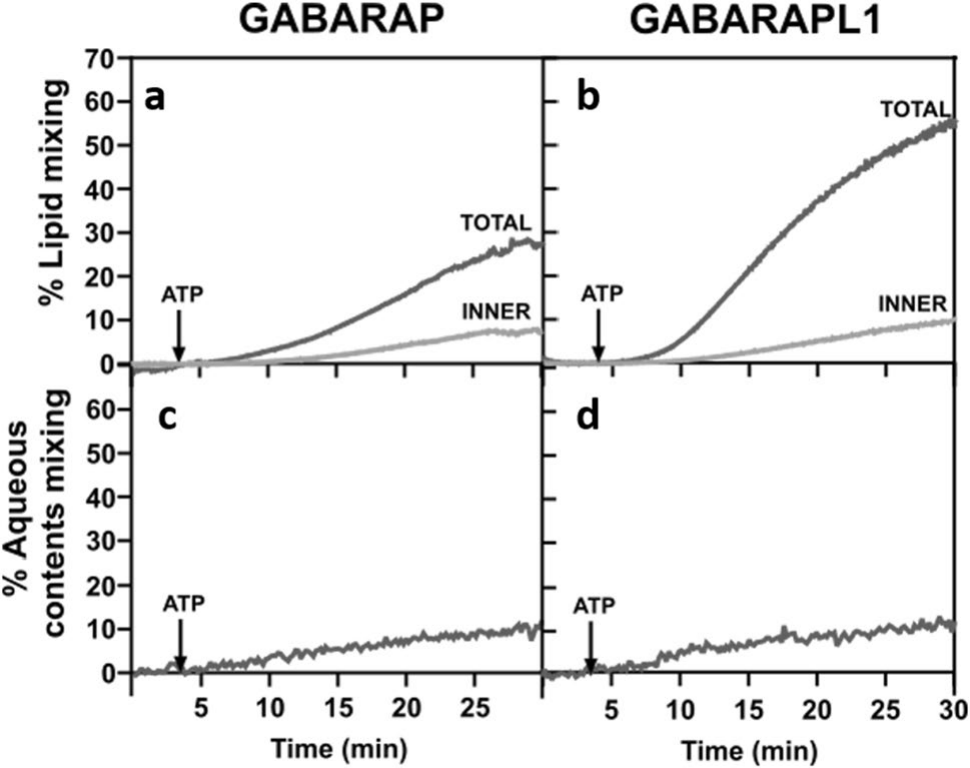
GABARAPL1 and GABARAP cause membrane hemifusion but are poor inducers of vesicle-vesicle fusion. **a**, **b** Representative curves of total (gray) and inner (light gray) lipid mixing activities by lipidated GABARAP (**a**) and GABARAPL1 (**b**) in the absence of the E3, monitored with the NBD-PE/Rho-PE lipid dilution assay. For inner monolayer lipid mixing, NBD/Rho-liposomes were pretreated with the appropriate amounts of sodium dithionite to quench NBD fluorescence of the outer leaflet. 0.4 mM of unlabeled and (NBD-PE + Rho-PE)-labeled liposomes (9:1) were mixed with 0.5 µM ATG7, 1 µM ATG3, and 5 µM of the pertinent LC3/GABARAP family member. After 4 min incubation, ATP was added. **c**, **d** Representative curves of aqueous contents mixing activities by lipidated GABARAP (**c**) and GABARAPL1 (**d**) in the absence of E3, monitored with the ANTS/DPX mixing assay. 0.4 mM ANTS and DPX liposomes (1:1) were mixed with 0.5 µM ATG7, 1 µM ATG3, and 5 µM of the pertinent LC3/GABARAP family member. After 4 min incubation, ATP was added. Co-encapsulated ANTS- and DPX-containing LUV were used to determine the 100% of aqueous contents mixing

The demonstration of vesicle-vesicle fusion requires the independent observation of vesicle tethering, total lipid mixing, inner-monolayer lipid mixing, and, in the absence of leakage, mixing of inter-vesicular aqueous contents [35, 46]. Since GABARAP and GABARAPL1 were the proteins showing a higher ability to induce total lipid mixing (Fig. 5d, e), we decided to explore if they were also able to induce lipid mixing of the vesicle inner monolayers, to determine whether the observed process was one of membrane hemifusion or of full fusion. The results indicated that, even if some inner lipid monolayer mixing occurred (Fig. 6a, b), the extent reached remained well below the 50% of the total lipid mixing required for an extensive fusion event. As expected from their low total lipid mixing levels, LC3C and GABARAPL2 did not elicit any sizeable signal of inner lipid mixing (data not shown).

To confirm these results, we next measured the ability of GABARAP and GABARAPL1 to produce fusion using an aqueous contents mixing assay. A preliminary check had to be performed to determine whether, once lipidated, LC3/GABARAP proteins induced the release of vesicular aqueous contents (leakage) or not (Supp. Fig. 10). No leakage was observed under our conditions, neither in the presence nor in the absence of E3, therefore, the aqueous contents mixing assay could be performed, providing meaningful results. As expected from the low levels of inner-monolayer lipid mixing, a low capacity of GABARAP or GABARAPL1 to produce aqueous contents mixing was recorded (Fig. 6c, d). Furthermore, in accordance with the E3 effect on GABARAP and GABARAPL1 lipid mixing ability, the small amount of aqueous contents mixing was totally abolished when E3 was present (Supp. Fig. 11a, b).

**Fig. 7:**
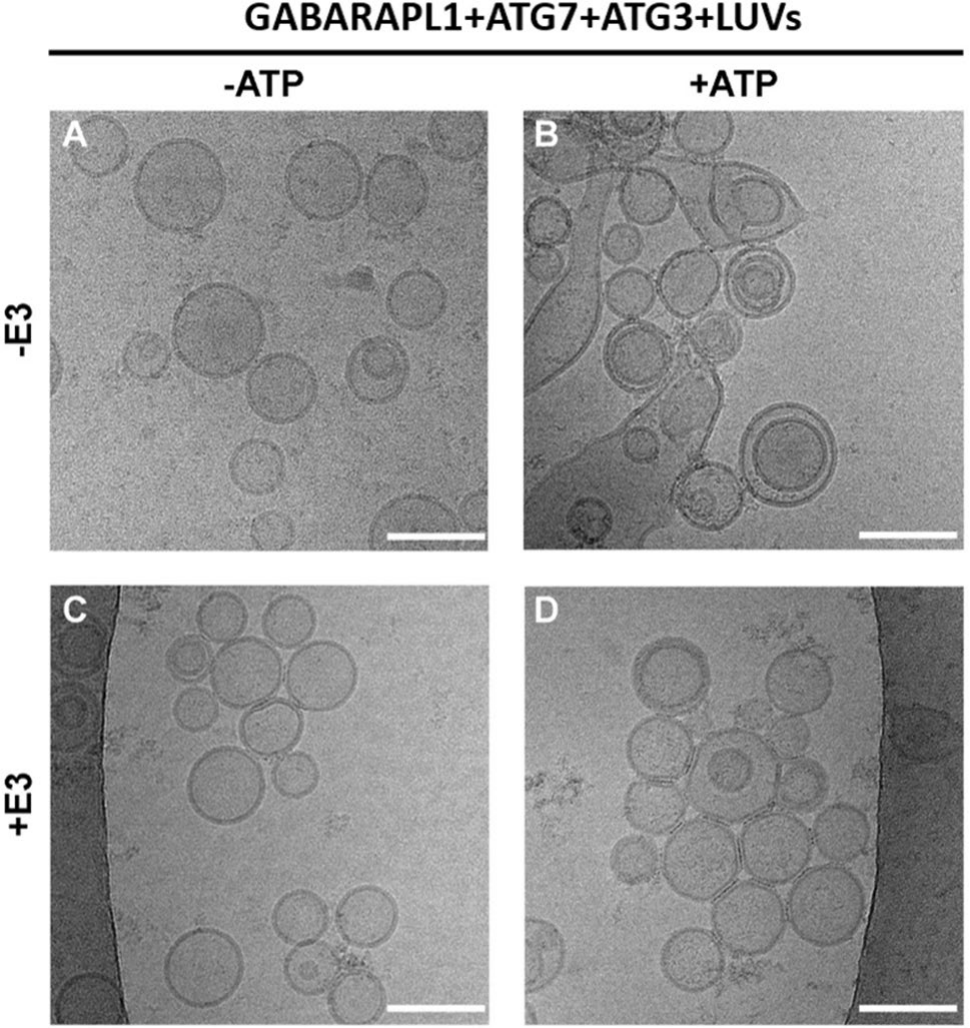
GABARAPL1 ability to tether and fuse vesicles in the absence and presence of E3 analyzed by cryo-EM. Cryo-EM images of the four conditions analyzed in Figs 4E and 5E. 0.5 µM ATG7, 1 µM ATG3, and 5 µM GABARAPL1 were mixed with 0.4 mM LUV [ePC:DOPE:PI:DOG (33:55:10:2 mol ratio)], in the absence (− E3) or in the presence (+ E3) of 0.1 µM E3. After addition of buffer (-ATP) or ATP (+ ATP), the mixture was incubated at 37 °C for 90 min. **a–d** Cryo-EM images of liposomes after reconstituting GABARAPL1 conjugation reaction: **a** in the absence of E3 and ATP, **b** in the absence of E3 but in the presence of ATP, **c** in the presence of E3 but in the absence of ATP and **d** in the presence of both E3 and ATP. Bar = 100 nm

Moreover, GABARAPL1 ability to cause vesicle tethering and hemifusion was analyzed using cryo-electron microscopy (Cryo-EM). In the absence of E3 and of ATP (Fig. 7a), the vesicles appeared well differentiated, mostly unilamellar, and with a diameter close to 80 nm. Addition of ATP, which induced GABARAPL1 lipidation, caused extensive vesicle tethering, with membrane contacts and some extended sheet-like structures, 300–400-nm long, (Fig. 7b) all of them compatible with a degree of membrane fusion. Supp. Fig. 12 displays several images of the condition “− E3, + ATP,” in which examples of structures evocative of aggregation and hemifusion (triple parallel lines and inter-vesicular discontinuous lines) and fusion (sheets) can be seen. This is in agreement with the spectroscopic data in Fig. 4e (gray solid line), Fig. 5e (gray solid line) and Fig. 6b, d. Cryo-EM of vesicles treated with E3, but not ATP, indicate only some vesicle tethering/aggregation (Fig. 7c), as expected from the turbidity data in Fig. 4e (green dashed line). Finally, vesicles in the presence of both ATP and E3 did show triple parallel lines as a signal of aggregation and a few inter-vesicular discontinuous lines, but without extended structures suggestive of fusion (Fig. 7d), again as expected from the fluorescence data (Fig. 5e, green solid line, and Supp. Fig. 11). Galleries of images obtained under the conditions in Fig. 7a–d can be found in Supp. Fig. 13. Thus, the overall results obtained suggest a mode of action of lipidated GABARAP and GABARAPL1 in the absence of E3 compatible with a large fraction of the vesicles undergoing close apposition, or hemifusion, and a minor fraction carrying out full fusion.

## Discussion

### Differences in LC3/GABARAP protein activities suggest the existence of a lipidation threshold, lower for the GABARAP subfamily

One of the main aims in this study was to assess E3 effect on the ability of LC3/GABARAP proteins to promote vesicle tethering and fusion. Our results pointed to two relevant observations, one was that, under otherwise similar conditions, E3-independent lipidation appeared to differ for each subfamily (Fig. 2a–f). GABARAP subfamily members were the most easily lipidated homologs. In turn, LC3A and LC3B reached low lipidation levels, but LC3C was the exception to the rule, see below (Fig. 2g, h, − E3 panel). These results agree with those by Lystad et al. [39], who showed that E3 was essential for LC3B lipidation, while the GABARAP subfamily was less E3-dependent, since it could be lipidated in the absence of E3 in liposomes under certain conditions. However, the inclusion of six family members in our study revealed that E3 effects did not strictly depend on the subfamilies. In particular, while LC3A behaved similarly to LC3B, LC3C could be lipidated to a large extent in the absence of E3 (Fig. 2c, g), thus parting with the rest of the LC3 subfamily. LC3C equally failed to follow the general trends of the LC3 subfamily in previous studies on cardiolipin-mediated mitophagy [26].

The second observation worthy of comment is the existence of a lag phase in the absence of E3 (Fig. 4h and Fig. 5h, − E3 panel), suggesting the need to reach a lipidation threshold before proceeding to deeper levels of interaction with the host lipid bilayer. The situation is reminiscent of the lag phase required by phospholipase C before inducing vesicle aggregation [47]. Taking into account that the growing edge of the phagophore should be a narrow area, with a high concentration of lipids with negative curvature but leaving little space for proteins, a protein that could induce membrane fusion with the minimum number of molecules per area would be needed. The tethering/lipidation or lipid mixing/lipidation ratios (Fig. 4i and Fig. 5i, − E3 panel) pointed to a lower lipidation threshold for all the GABARAP proteins as compared to the LC3 subfamily, suggesting that members of the GABARAP family would be excellent candidates to perform this function.

### The E3-induced increase in lipidation enhanced and accelerated LC3/GABARAP-promoted vesicle tethering but reduced their lipid mixing ability

The interaction of E3 with membranes of different composition and curvature has been recently described, showing that ATG16L1 is the main protein responsible for E3 interaction with membranes, both in human [39, 48] and yeast proteins [49]. However, these studies did not consider the effect of E3 on vesicle tethering, detected in yeast by Romanov et al. [11]. Under our experimental conditions, with lower protein concentration and smaller curvature, E3 caused no aggregation on its own (Fig. 3a). However, the presence of ATG3 elicited membrane tethering, albeit to a low extent. (Fig. 3b). This positive effect could be explained by the well-known interaction between ATG12 and ATG3 [50–52]. Such an interaction could increase E3 affinity toward the membrane (Fig. 3c), thus the activation of E3-dependent tethering activity. This effect could also be a combination of both proteins, as an ATG3-dependent tethering activity sensitive to lipid composition was already shown [53]. The E3-promoted conformational change in ATG3 [54] could also activate its tethering activity and make ATG3 act in combination with E3, however, further studies would be needed to understand this behavior. In any case, this initial aggregation of vesicles could be partially responsible for the faster lipidation and tethering effects seen once ATP was added (Fig. 4a–f).

Including E3 in our in vitro system was aimed at getting the six members of the family lipidated to > 50% and to about the same extent in all cases. This made possible the comparison of LC3/GABARAP proteins, at similar levels of lipidation, in their ability to induce tethering and fusion of membranes. Such lipidation levels were achieved with low amounts of E3 (Fig. 1b). The presence of E3 accelerated and increased lipidation, reaching levels of at least 70% in 30 min under our conditions (Fig. 2g, + E3 panel). Note that, when E3-enhanced lipidation rates are compared, LC3C and GABARAPL1 continue to be the fastest ones in being lipidated (Fig. 2h, + E3 panel), just as they were in the absence of E3.

When E3 was present, lipidation levels of LC3/GABARAP proteins were also related to their tethering ability. Their increased lipidation allowed the participation of any of the LC3/GABARAP members in aggregation events (Fig. 4a–f). The absence of a lag phase when E3 was present (Fig. 4h) suggested that under those conditions, all the proteins were able to reach their lipidation threshold earlier. E3 interaction with membranes and the subsequent vesicle aggregation (Fig. 3), together with the positive effect of ATG3, could explain the acceleration. Moreover, comparing the lipidated LC3/GABARAP protein tethering activities and relating them to the protein lipidation levels reached during those experiments, no differences among the different family members were observed when E3 was present (Fig. 4i). Thus, E3 can equalize the level of lipidation of the various LC3/GABARAP family members and therefore their capacities to cause membrane tethering. However, E3 did not have the same effect on all proteins when it came to inducing inter-vesicular lipid mixing. E3 clearly lowered the lipid mixing activity of the two most active proteins in this respect, GABARAP and particularly GABARAPL1 (Fig. 5d, e). The outstanding questions are why E3 decreases their ability to produce inter-vesicular lipid mixing, and why proteins with similar lipidation levels induce similar tethering but different levels of inter-vesicular lipid mixing.

In yeast, E3 had been detected on the convex face of the growing phagophore, together with Atg8, while in the concave face, only some Atg8-PE remained [55–57]. In this context, Kaufmann et al. [58] observed that, once the yeast Atg8 had been lipidated, it was able to associate with E3 into a membrane scaffold thanks to an Atg8-interacting motif (AIM) in Atg12. Although formation of such a scaffold with the different LC3/GABARAP proteins has not been described, it is conceivable that in our in vitro system, once the LC3/GABARAP proteins had reached a certain lipidation level, the scaffold would form on the liposome. The joint localization of E3 and GABARAPL1 assessed by the liposome flotation assay after lipidation (Supp. Fig. 3, + ATP panel) suggests the presence of a dense protein coat on the liposomes that could be compatible with the possibility of this scaffold being built.

**Fig. 8:**
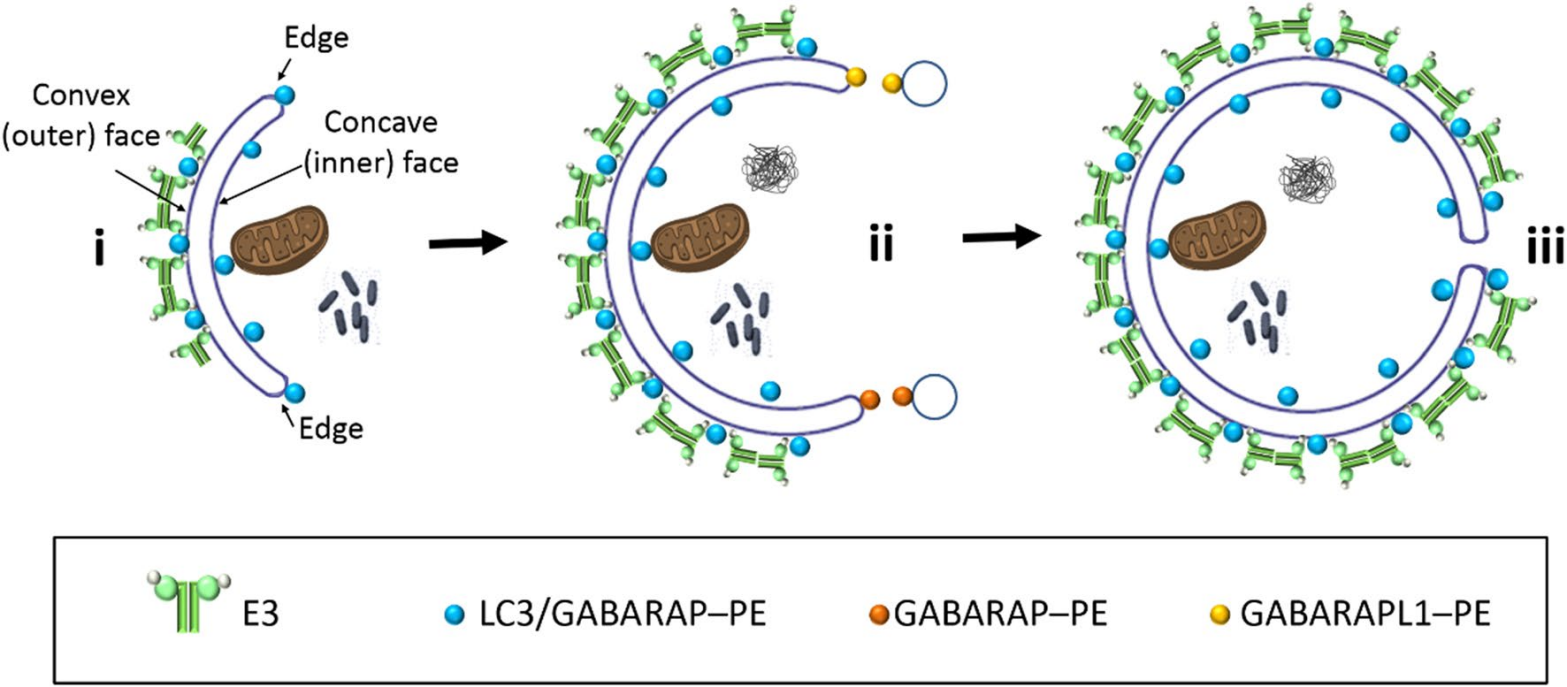
Role of the LC3/GABARAP proteins and E3 in the phagophore expansion process: a hypothetical model based on the results in this work. (i) LC3/GABARAP–PE is distributed along the whole phagophore surface. E3 could form an immobilescaffold with lipidated LC3/GABARAP proteins on the convex side of the outer bilayer [58], but not on the edges and growing zones of the phagophore. (ii) GABARAP and GABARAPL1 are the main candidates to promote the phagophore expansion, particularly on the highly curved edges, as these proteins reach faster the necessary lipidation levels to trigger vesicle tethering and inter-vesicular lipid mixing. (iii) The subsequent vesicle fusion mediated by the tethering and lipid mixing ability of these proteins (with the concerted action of other factors and proteins) will cause the expansion of the phagophore. See main text for details

Formation of such a scaffold could explain why GABARAP and GABARAPL1 had a lower lipid mixing ability when E3 was present (Supp. Fig. 8c): The scaffold would facilitate vesicle tethering but it would also hamper inter-vesicular lipid mixing, for which vesicle hemifusion or close apposition would be required [59–61]. The fact that the decrease was more marked for GABARAPL1 could indicate that more lipidated protein meant a larger scaffold formation and therefore a less favorable situation for lipid mixing. The hampered lipid mixing would have as an inevitable consequence the near-complete lack of fusion structures, as seen by cryo-EM (Fig. 7 and Supp. Fig. 13). The results would be compatible with the hypothesis that E3 could only form an immobile scaffold on the convex face of the growing AP [6, 58, 62]. However, its formation would not happen on the concave face, nor on the edges of the nascent AP, in order to allow successive rounds of vesicle fusion and therefore phagophore growth (Fig. 8).

### The role of GABARAP and GABARAPL1 in the phagophore expansion process: an evolutionary discussion

LC3/GABARAP proteins play different roles in autophagy. Their binding to autophagic receptors containing LIR motifs [22] is well known. Moreover, the LC3/GABARAP protein family is deemed very important in phagophore expansion [33]. Studies with knockouts of all six members of the family found that the autophagy mechanism could work in the absence of LC3/GABARAP proteins, although autophagosomes were formed at a much slower rate, they were smaller, and often had trouble fusing with lysosomes [63]. This points to an important, if not essential, role of LC3/GABARAP family in phagophore expansion.

Under our conditions, GABARAP and GABARAPL1 were the ATG8 family members promoting the most extensive vesicle tethering (Fig. 4d, e) and inter-vesicular lipid mixing (Fig. 5d, e). For these two proteins, lipid mixing included some degree of inner monolayer mixing and a low amount of aqueous contents mixing (Fig. 6). The scenario is one of vesicle hemifusion with occasional fusion events. Cryo-EM results were in accordance with the fluorescence spectroscopy results (Fig. 7). A more extensive fusion would require the localized presence (perhaps in nanodomains) of lipids with an intrinsic negative lipid curvature such as diacilgycerol or cardiolipin [35, 36] or the action of additional proteins in the growing areas of the phagophore.

Although this work is focused on the role that LC3/GABARAP proteins and their conjugation system may have in phagophore expansion, it should be mentioned that this model is not incompatible with other proteins participating in this process, such as ATG9 or ATG2 [64, 65]. ATG9-containing vesicles participate in several steps of AP formation, and ATG9 is proposed to aid expansion by fusing these vesicles with the phagophore [66, 67]. In the case of ATG2, it has been seen that it can transport lipids from the ER to the phagophore, providing part of the lipids necessary for this expansion [68]. It has recently been described that ATG2 and ATG9 can form a complex to act in a coordinated way [69]. The precise contribution of each of these three systems (LC3/GABARAP, ATG9 and ATG2) to the expansion process is still unknown, as is any putative kind of interaction between them, these aspects should be the object of future studies. For example, recent data have shown that the direct interaction between ATG2 and GABARAP/GABARAPL1 could be crucial for the correct formation and closure of the autophagosome [70].

In general, the above results show that the GABARAP subfamily is clearly more active than its LC3 homologs in the induction of membrane fusion. Since Atg8 in yeast has the ability to cause vesicle hemifusion [15], LC3s appear to have lost this function during evolution. This is consistent with GABARAPs being more evolutionarily related to Atg8 than LC3. The LC3 subfamily may have become more specialized in the recognition of autophagic receptors and adapters [22], losing functions related to vesicle fusion induction in the process. This hypothesis is consistent with the study performed with the Atg8 orthologs in *C. elegans* LGG-1 and LGG-2 [71]. Those authors found that the LGG-1 homolog, more similar to GABARAP, had the ability to tether and fuse vesicles, while LGG-2 (more similar to LC3) had only a limited capacity to induce tethering and none to fuse vesicles.

The hypothesis of the LC3 loss of fusogenic function along evolution can also help understand the results obtained in different studies with knockouts of the entire human ATG8 family, in which expressing GABARAP in ATG8-depleted cells leads to the recovery of autophagy, while LC3 expression does not [72], and the expression of LC3s can actually have a negative effect on autophagy [73]. It is possible that LC3, lacking the vesicle fusion activity, cannot replace the absence of GABARAP, while the latter, possessing a fusogenic activity and with the ability to recognize LIR sequences, can almost fully replace the LC3 functions. LC3C is an exception to this model but as this homolog is evolutionarily more distant [34], it could follow a different regulation pattern.

## Concluding remarks

Assaying protein lipidation, vesicle tethering and inter-vesicular lipid mixing activities of all members of the LC3/GABARAP family under the same experimental conditions allow a number of conclusions to be drawn. (i) While the large differences between GABARAPL1/GABARAP and LC3A/LC3B resemble the ‘canonical’ differences between the two subfamilies shown in other studies, LC3C appears as an unusual case within the LC3 subfamily, with a tethering activity akin to the one of the GABARAP subfamily. (ii) GABARAP and GABARAPL1 appear to be the most efficient homologs in the entire family for vesicle tethering and lipid mixing. However, as they are able to produce but a low level of full fusion, other proteins or the presence of other lipids that promote fusion could be needed in the in vivo situation. (iii) The results suggest a model in which the growing regions of the phagophore would be highly bent areas, at the phagophore edge, containing lipids with a negative intrinsic curvature, compatible with points of membrane fusion. In those regions, some of the LC3/GABARAP proteins could be lipidated without E3, or in the case that E3 helped lipidation, a regulation should exist to allow fusion of vesicles in those regions and induce phagophore expansion. (iv) The fact that LC3A or LC3B showed more difficulties to be lipidated even in the presence of E3 points to other functions for these homologs during autophagy, such as cargo receptors.

### Supplementary Information

Below is the link to the electronic supplementary material.

Supplementary file1 (DOCX 3727 kb)

## Data Availability

Data will be made available upon reasonable request.

## Abbreviations

ANTS: 8-Aminonaphthalene-1,3,6-trisulfonic acid, disodium salt
AP: Autophagosome
Atg8: Autophagy-related 8
ATP: Adenosine triphosphate
Cryo-EM: Cryo-electron microscopy
DOG: Egg dioleoylglycerol
DOPE: 1,2-Dioleoyl-*sn*- glycero-3-phosphatidylethanolamine
DPX: *p*-Xylene-bis-pyridinium bromide
DTT: DL-dithiothreitol
E3: E3-like complex (ATG12–ATG5-ATG16L1)
ePC: L-α- phosphatidylcholine from hen egg yolk
GABARAP: GABA type A receptor-associated protein
GABARAPL1: GABA type A receptor-associated protein like 1
GABARAPL2: GABA type A receptor-associated protein like 2
LUV: Large unilamellar vesicle/s
MAP1LC3A/LC3A: Microtubule-associated protein 1 light chain 3alpha
MAP1LC3B/LC3B: Microtubule-associated protein 1 light chain 3 beta
MAP1LC3C/LC3C: Microtubule-associated protein 1 light chain 3 gamma
NBD-PE: N-(7-nitrobenz-2-oxa-1,3-diazol-4-yl)-1,2-dihexadecanoyl-*sn*-glycero-3-phosphatidylethanolamine
NBDtail-PE: 1-Oleoyl-2-{6-[(7-nitro-2-1,3-benzoxadiazol-4-yl) amino] hexanoyl}-*sn*-glycero-3-phosphoethanolamine
PE: Phosphatidylethanolamine
PI: Liver phosphatidylinositol
Rho-PE: 1,2-Dioleoyl-*sn*-glycero-3-phosphatidylethanolamine-N-lissamine rhodamine B sulfonyl
UBL: Ubiquitin-like
