# Supplementary material for: Effect of ATG12–ATG5-ATG16L1 autophagy E3-like complex on the ability of LC3/GABARAP proteins to induce vesicle tethering and fusion
Source: Cell Mol Life Sci. 2023 Feb 2;80(2):56. doi: 10.1007/s00018-023-04704-z (PMC9894987; doi:10.1007/s00018-023-04704-z)

**Supplementary Information for**

**Effect of ATG12–ATG5-ATG16L1 autophagy E3-like complex on the ability of LC3/GABARAP proteins to induce vesicle tethering and fusion**

Marina N. Iriondo^1,2^, Asier Etxaniz^1,2^, Yaiza R. Varela^1,2^, Uxue Ballesteros^1,2^, Melisa Lázaro^3^, Mikel Valle^3^, Dorotea Fracchiolla^4^, Sascha Martens^4^, L. Ruth Montes^1,2^, Félix M. Goñi^1,2^, Alicia Alonso^1,2&^

**This file includes:**

Supp. Fig. 1-13

Supp. Table 1


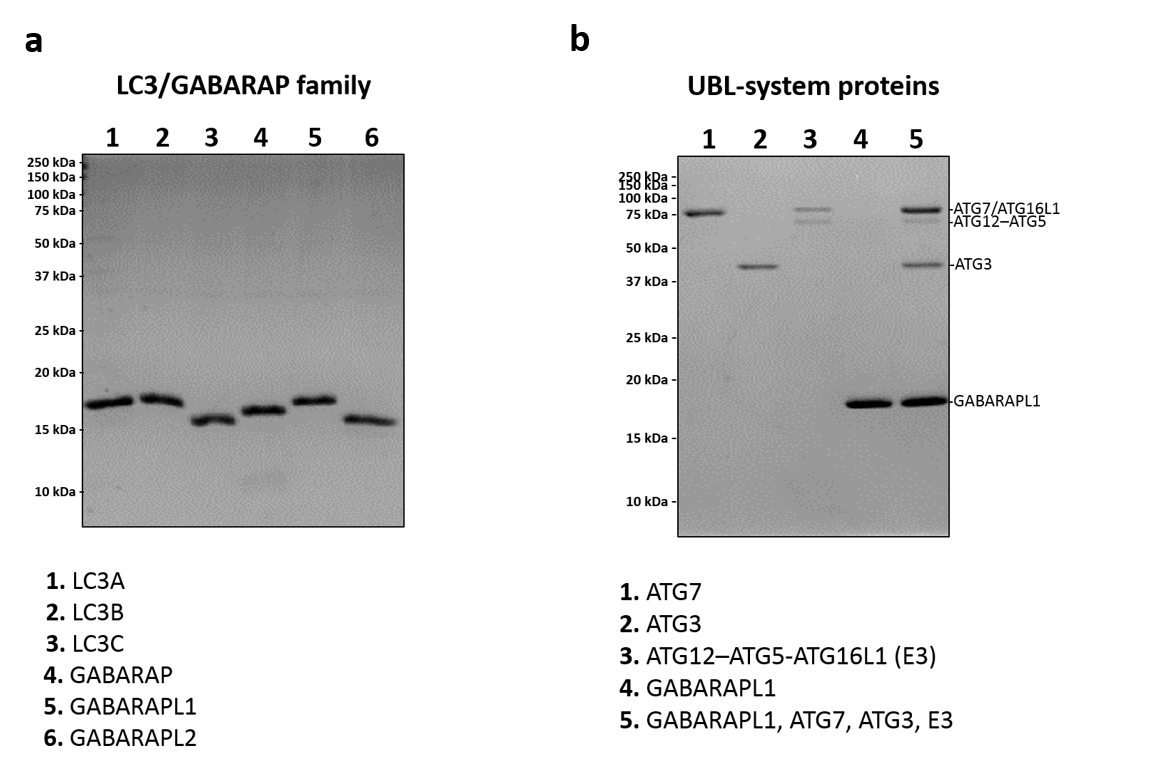


**Supp. Fig. 1**. LC3/GABARAP protein family and UBL-system proteins. **a.** LC3/GABARAP protein family. Each family member (5 µM) was loaded on a SDS-PAGE gel and stained with Coomassie Brilliant Blue. The one exhibiting a smaller electrophoretic mobility was GABARAPL1, followed by LC3B, LC3A, GABARAP, GABARAPL2 and LC3C. **b.** UBL-like system proteins. Each protein was loaded at assay concentration (0.5 µM ATG7 | 1 µM ATG3 | 0.1 µM E3 | 5 µM GABARAPL1) on a SDS-PAGE gel and stained with Coomassie Brilliant Blue. Lane 5 contains a mixture of the four proteins, showing the overlapping between ATG7 and ATG16L1.


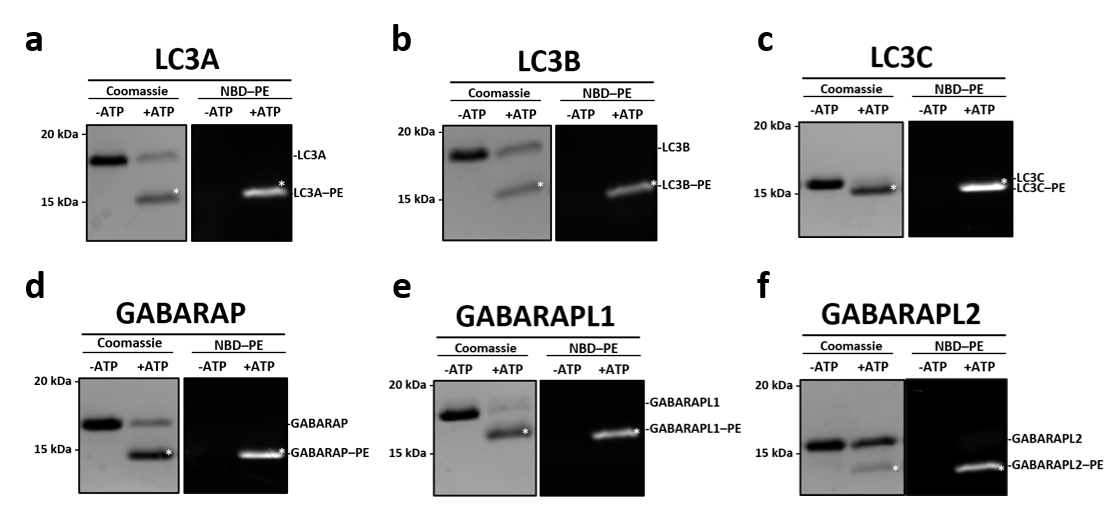


**Supp. Fig. 2.** The faster migrating band observed after ATP addition represents the lipidated LC3/GABARAP protein. **a-f** *In vitro* LC3/GABARAP lipidation assay: 0.5 µM ATG7, 1 µM ATG3, 0.1 µM E3 and 5 µM of the indicated LC3/GABARAP-protein member were mixed with 0.4 mM LUVs (ePC:DOPE:PI:DOG:NBDtail-PE (33:45:10:2:10 mol ratio)). Each reaction mixture was incubated at 37°C for 30 min, run on 15% SDS-PAGE gels, and visualized by Commassie Brilliant Blue staining (left panels) or using a VersaDoc MP 4000 Imaging System to detect NBD-PE fluorescence (right panels). Only the faster migrating band (*) showed fluorescence.


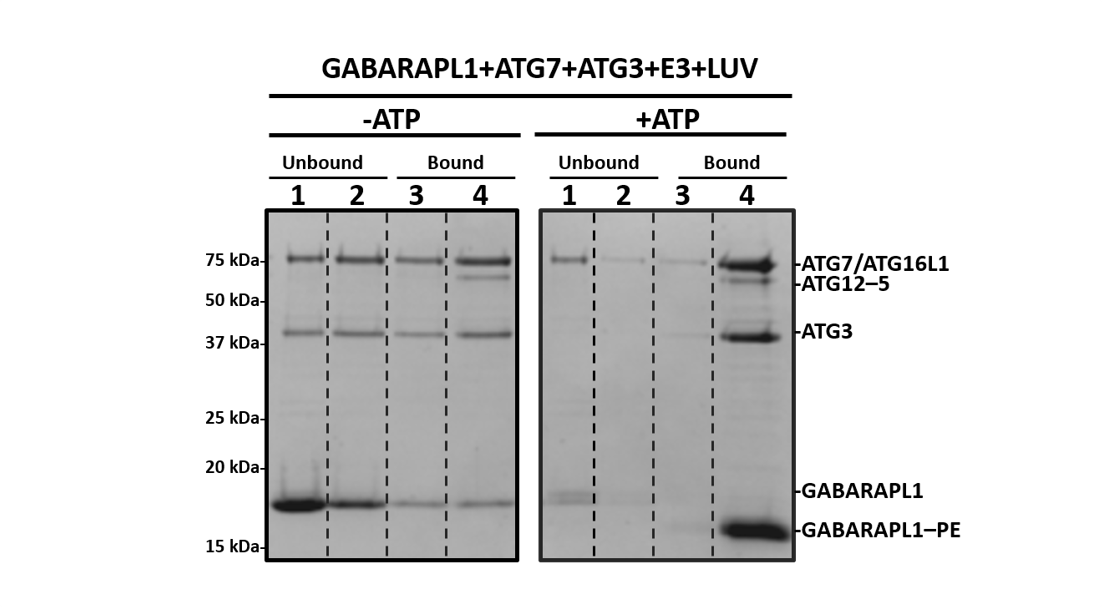


**Supp. Fig. 3.** Membrane interaction of the lipidation machinery increases in the presence of ATP. SDS-PAGE/Coomassie Brilliant Blue-stained gels of the fractions obtained from a vesicle flotation assay of the proteins that form the lipidation machinery in the absence (left) or presence (right) of ATP. Protein found in fractions 3+4 was taken as bound protein. Protein and lipid concentrations were increased by 5-fold in order to detect E3 in the gels. 2.5 µM ATG7, 5 µM ATG3, 0.5 µM E3 and 25 µM of GABARAPL1 were incubated with 2 mM LUVs (ePC:DOPE:PI:DOG (33:55:10:2 mol ratio)) at 37°C for 30 min in the absence or presence of ATP.


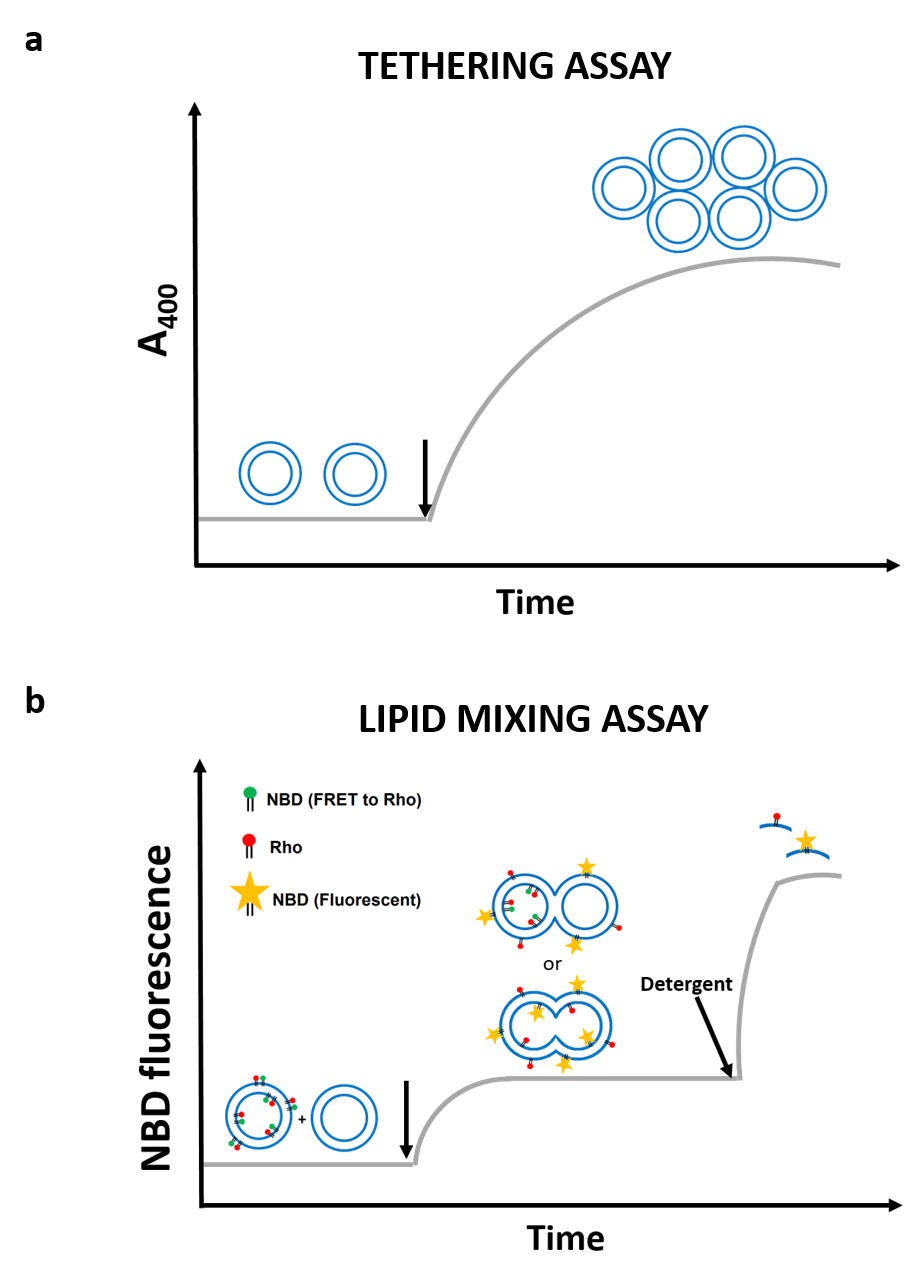


**Supp. Fig. 4.** An outline of the tethering and lipid mixing assays. **a** Vesicle tethering assay: Initially vesicles scarcely scatter light. Upon addition of a tethering-inducing agent (arrow), aggregation occurs and turbidity increases, thus light scattering increases in parallel. **b** Inter-vesicle lipid mixing assay: Initially there are two populations of vesicles, one labelled with the FRET pair and the second unlabelled. Upon addition of a tethering-inducing agent (first arrow), if lipid mixing occurs, the energy transferred between NBD and rhodamine decreases, therefore NBD fluorescence increases. A detergent (second arrow) is added to achieve complete vesicle solubilization; under these conditions, 100% NBD fluorescence value is obtained.


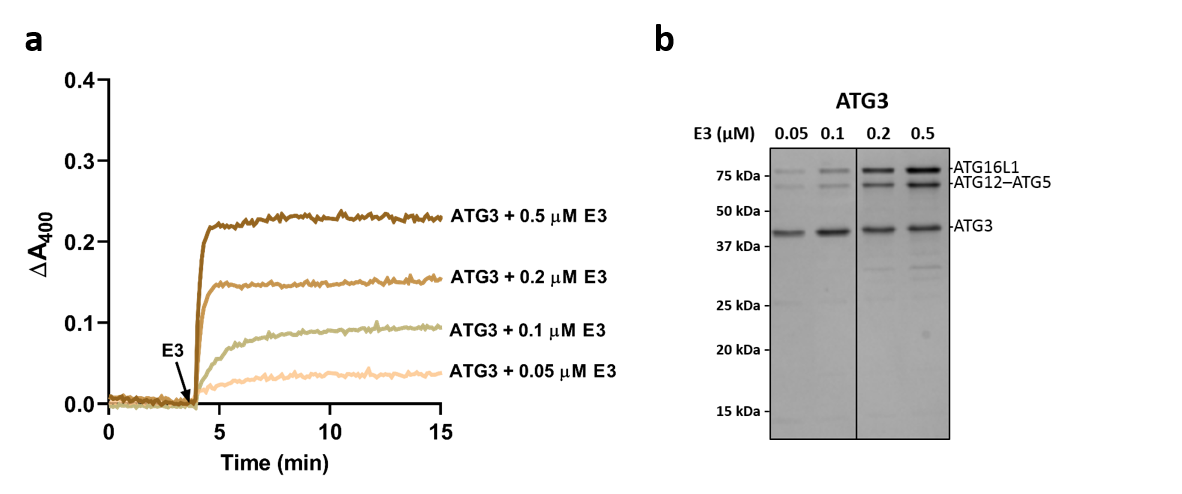


**Supp. Fig. 5.** Tethering levels observed in the presence of ATG3 upon E3 addition are E3-concentration dependent. **a**. Changes in turbidity (ΔA_400_), as a signal of vesicle tethering, were measured after E3 addition. Tethering of 0.4 mM LUV [ePC:DOPE:PI:DOG (33:55:10:2 mol ratio)] caused by addition of 0.05, 0.1, 0.2 and 0.5 µM E3 in the presence of 1 µM ATG3. **b**. SDS-PAGE gels of each condition visualized by Coomassie Brilliant Blue staining.


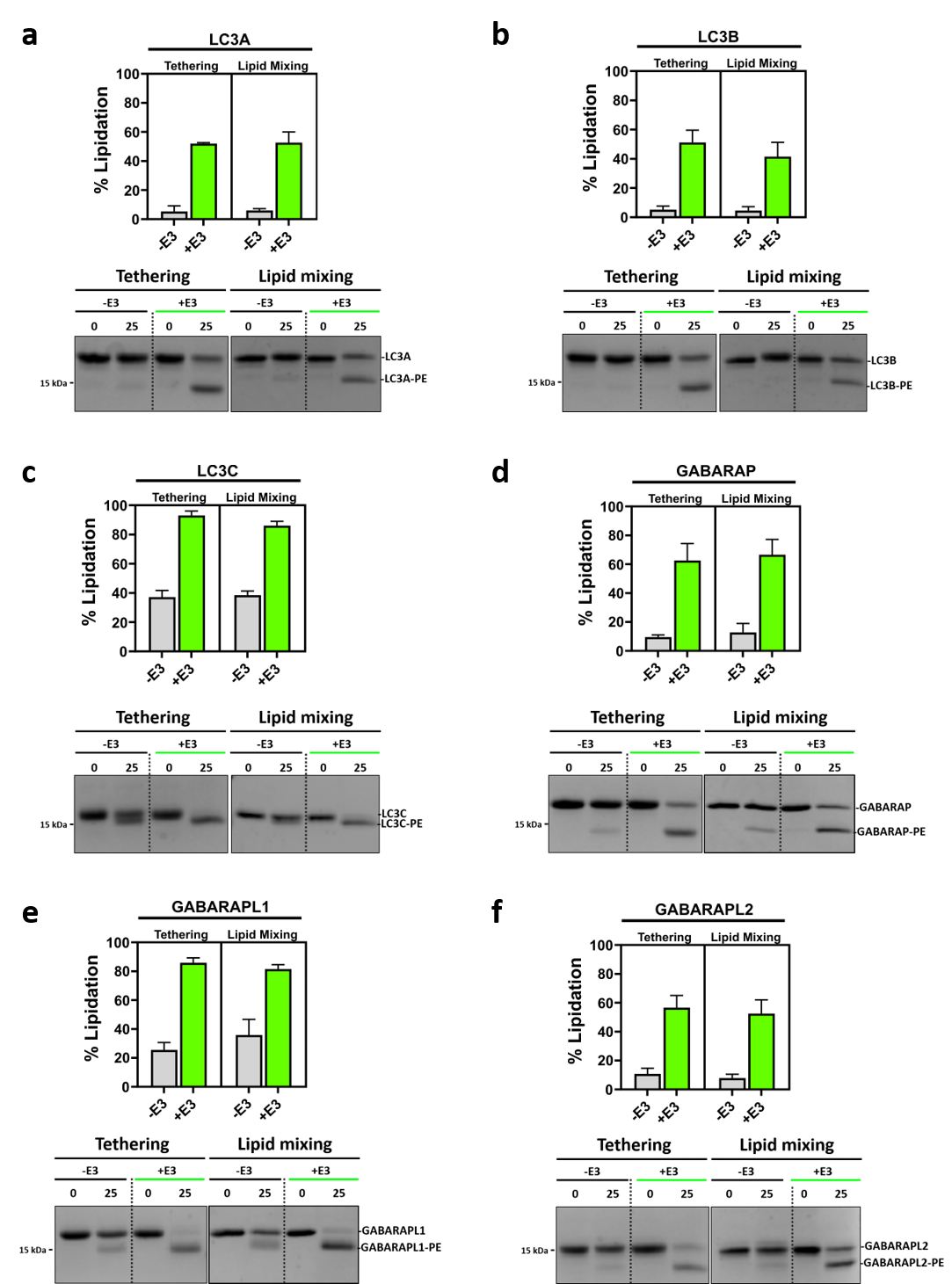


**Supp. Fig. 6.** Lipidation of LC3/GABARAP proteins during tethering and lipid mixing assays**.** **a-f** Top panels: Final lipidation levels in the absence (grey) and presence (green) of E3. Data are means ± SD (n = 3). Bottom panels: Representative gels of the lipidation of each protein during the tethering and lipid mixing assays before and after 25 min ATP addition in the presence and in the absence of E3.


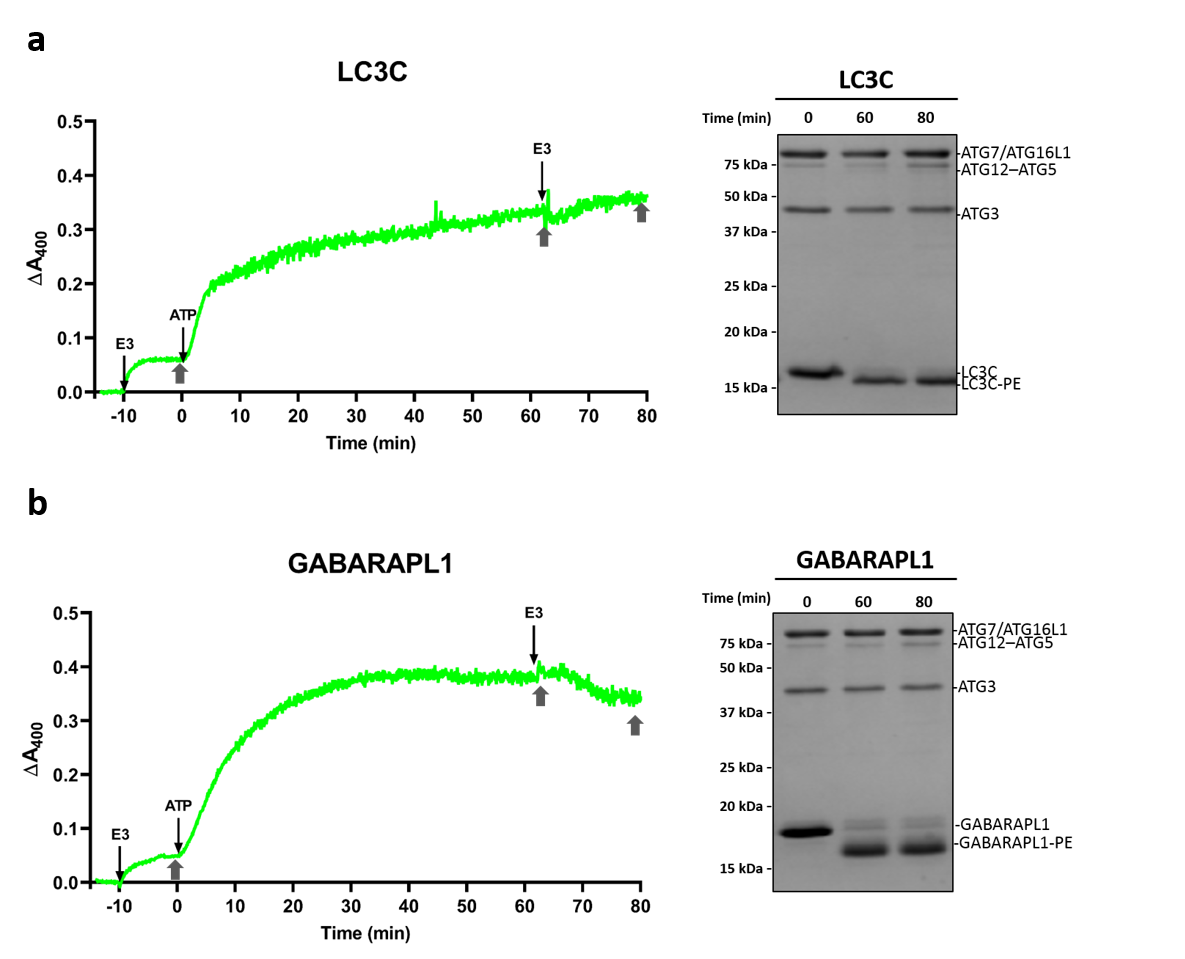


**Supp. Fig. 7.** E3-like complex addition did not promote higher tethering levels when the protein was already fully lipidated. 0.4 mM LUV [ePC:DOPE:PI:DOG (33:55:10:2 mol ratio)], 0.5 µM ATG7, 1 µM ATG3, and 5 µM of the pertinent LC3/GABARAP-family member were mixed. After 4 min, 0.1 µM E3-like complex was added, and 10 min later ATP. 60 min after ATP addition, when all the protein was lipidated, 0.1 µM E3-like complex was added. Changes in absorbance at 400 nm (ΔA_400_), as an indication of vesicle tethering, were measured. The time at which SDS-PAGE samples were taken is indicated by the thick arrows. a. Representative curve and SDS-PAGE gel of LC3C. b. Representative curve and SDS-PAGE gel of GABARAPL1.


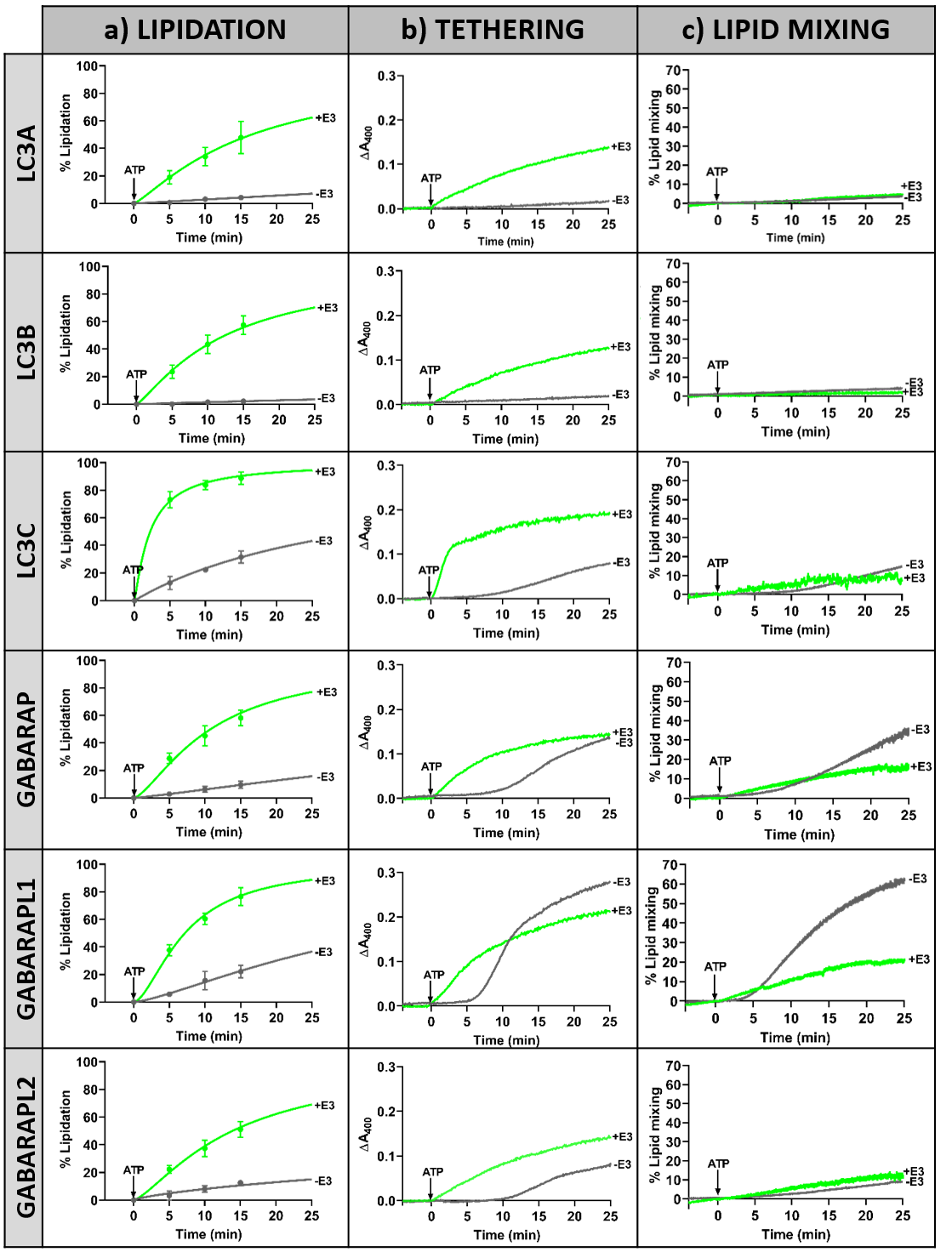


**Supp. Fig. 8.** Comparative summary of LC3/GABARAP-protein lipidation, tethering and lipid mixing kinetics in the absence and presence of E3. Data from Figure 2, Figure 4 and Figure 5 were redrawn in order to allow an easier comparison of the results in this study. **a** Lipidation kinetics of LC3/GABARAP proteins after ATP addition in the absence (grey) and in the presence (green) of E3. **b** Tethering kinetics of each LC3/GABARAP-family member after ATP addition in the absence (grey) and in the presence (green) of E3. **c** Lipid mixing kinetics of LC3/GABARAP proteins after ATP addition in the absence (grey) and in the presence (green) of E3.


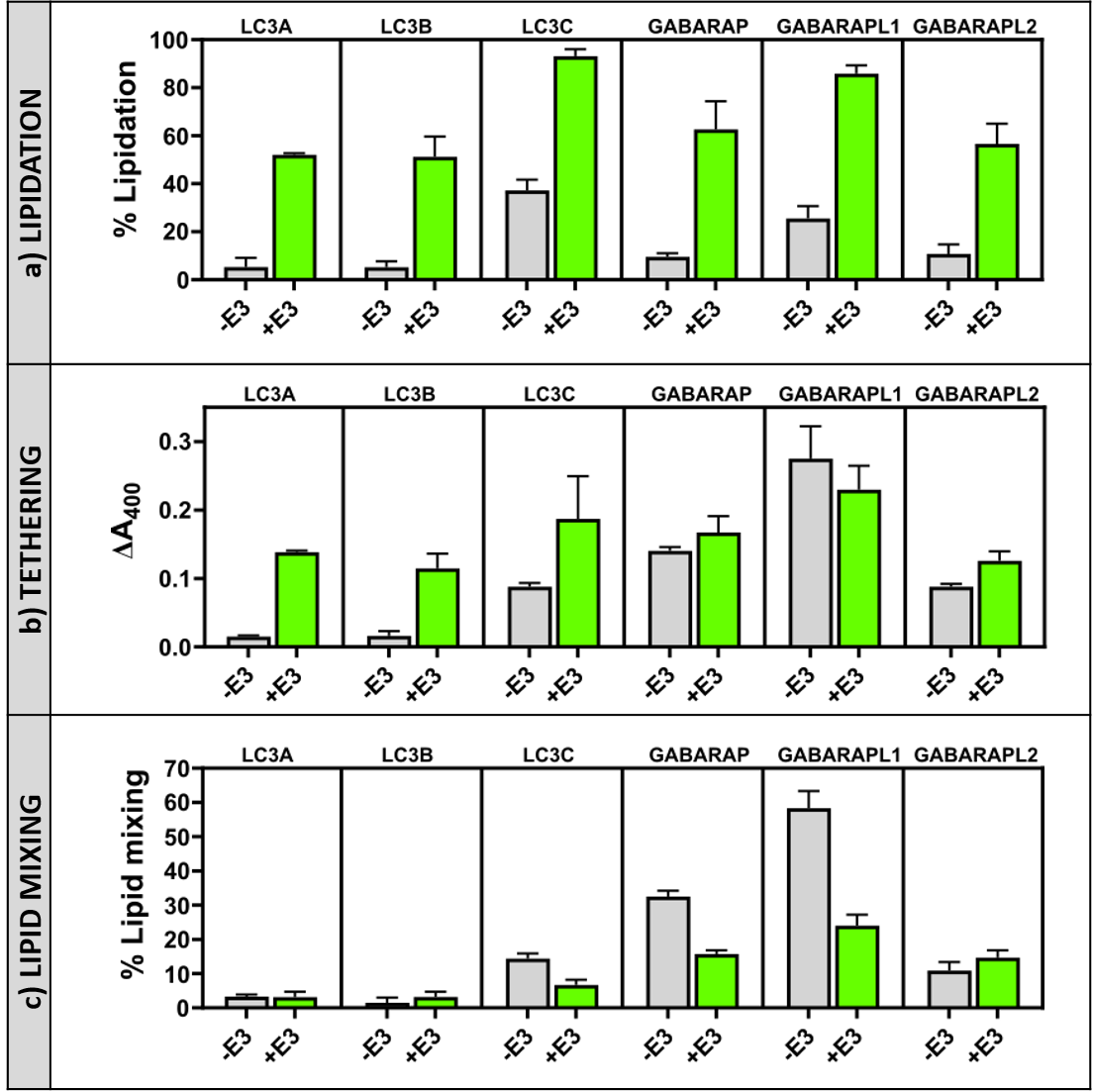


**Supp. Fig. 9.** Effect of E3 on LC3/GABARAP-protein lipidation and the subsequent vesicle tethering and lipid mixing 25 min after ATP addition. **a** Final lipidation levels obtained after the tethering assay (similar to the ones obtained after lipid mixing, see Supp. Fig. 6) in the absence (grey) and presence (green) of E3. **b** Extent of vesicle tethering induced by LC3/GABARAP proteins in the absence (grey) and presence (green) of E3. **c** Extent of lipid mixing induced by LC3/GABARAP proteins in the absence (grey) and presence (green) of E3. Data are means ± SD (n = 3).


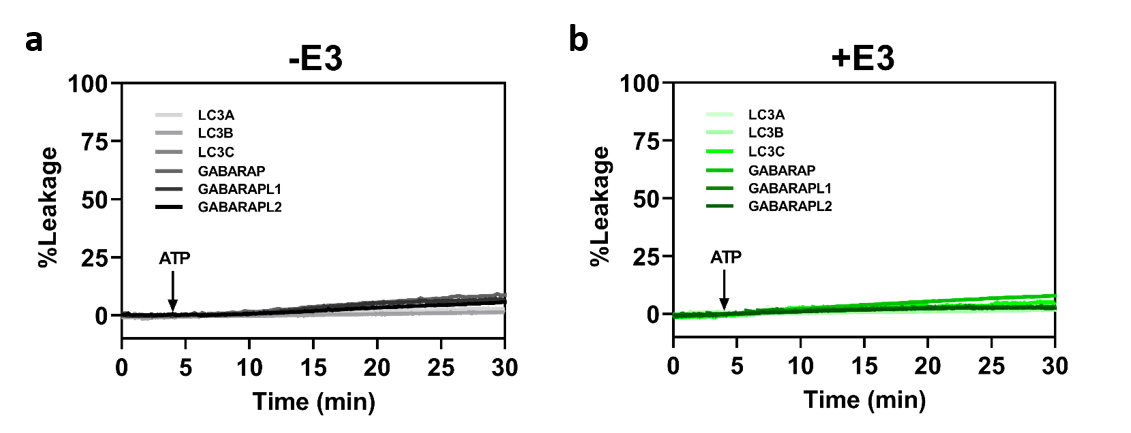


**Supp. Fig. 10.** Lipidated LC3/GABARAP proteins did not induce vesicle permeabilization neither in the absence nor in the presence of E3. Membrane vesicle content leakage analysis induced by lipidated LC3/GABARAP proteins in the absence and presence of E3 was monitored by the ANTS/DPX leakage assay. 0.4 mM LUVs containing co-encapsulated ANTS and DPX were mixed with 0.5 µM ATG7, 1 µM ATG3, and 5 µM of the pertinent LC3/GABARAP-family member and ATP was added after 4 min. 100% leakage signal was obtained by adding 1% Triton X-100. Contents leakage in the absence (a) or in the presence (b) of E3.


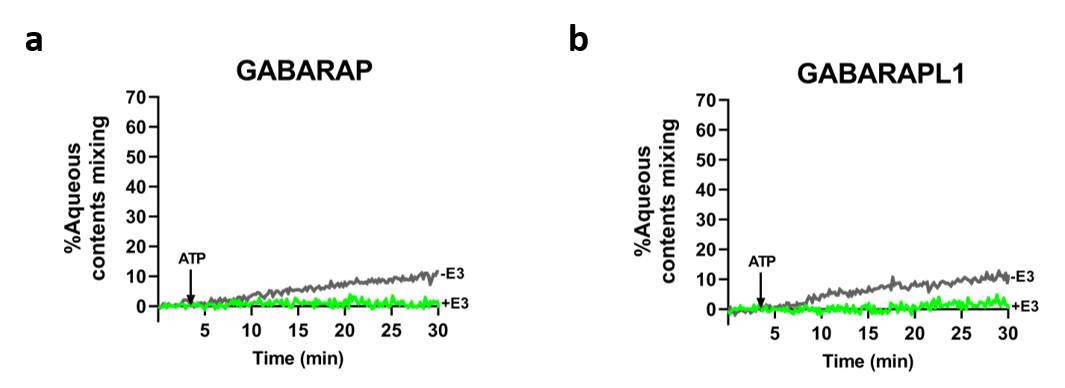


**Supp. Fig. 11.** E3 decreases the low aqueous content mixing activity of GABARAP and GABARAPL1. Representative curves of aqueous contents mixing activities induced by lipidated GABARAP (a) and GABARAPL1 (b) in the absence (-E3, grey) and presence (+E3, green) of E3 was monitored by the ANTS/DPX mixing assay. 0.4 mM ANTS and DPX liposomes (1:1) were mixed with 0.5 µM ATG7, 1 µM ATG3, and 5 µM of the pertinent LC3/GABARAP-family member, ATP was added after 4 min. 100% mixing was determined using LUVs containing co-encapsulated ANTS and DPX.


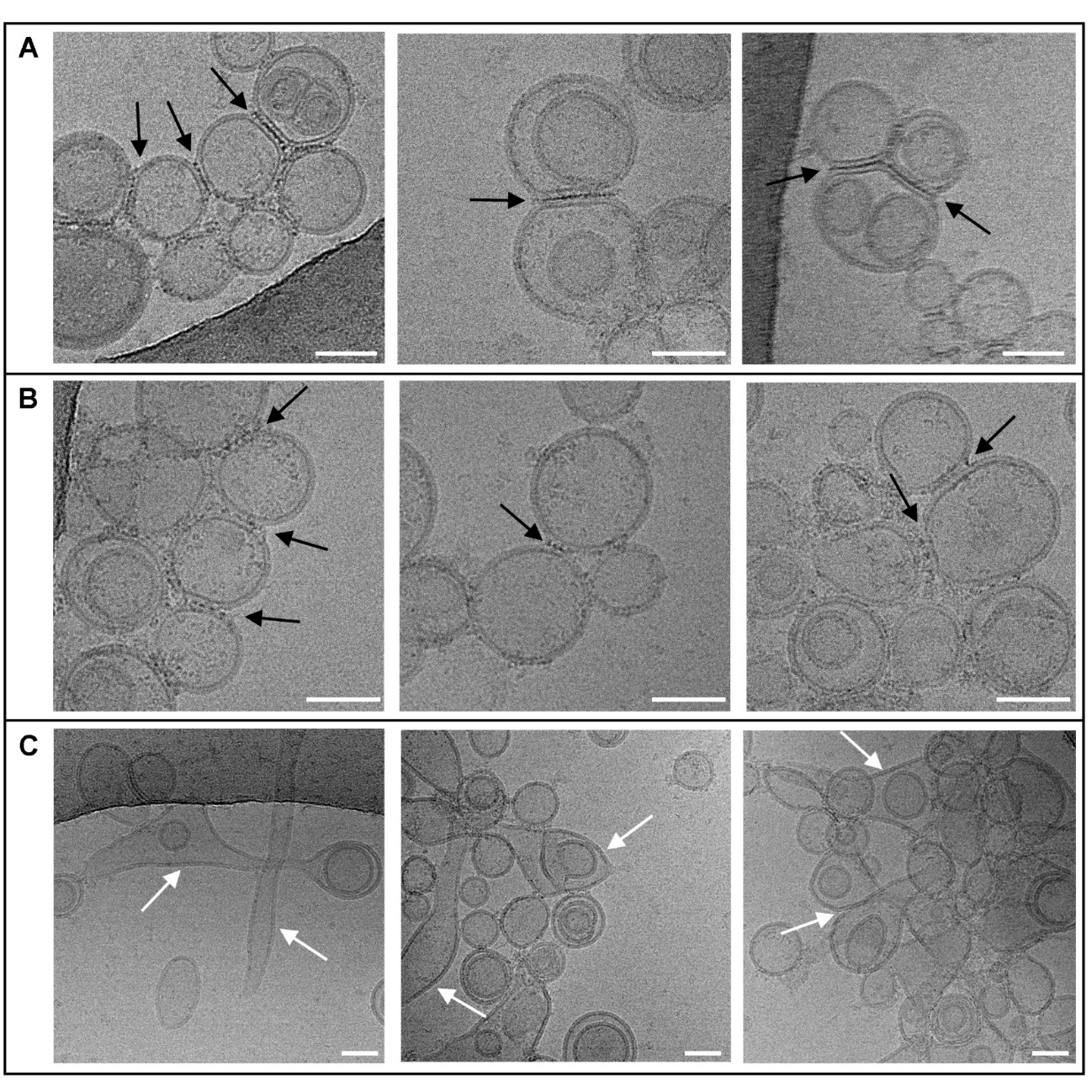


**Supp. Fig. 12.** GABARAPL1 ability to tether and fuse vesicles in the absence of E3, analyzed by cryo-EM. A gallery of cryo-EM images obtained under the condition “-E3, +ATP”. 0.5 µM ATG7, 1 µM ATG3, and 5 µM GABARAPL1 were mixed with 0.4 mM LUVs (ePC:DOPE:PI:DOG (33:55:10:2 mol ratio)) and, after ATP addition, they were incubated at 37°C for 90 min. Arrows point to the specific structures observed. **a** Examples of structures evocative of aggregation or hemifusion (“triple parallel lines”). **b** Examples of structures evocative of hemifusion (intervesicular discontinuous lines). **c** Examples of structures evocative of fusion (sheets). Bar = 50 nm.


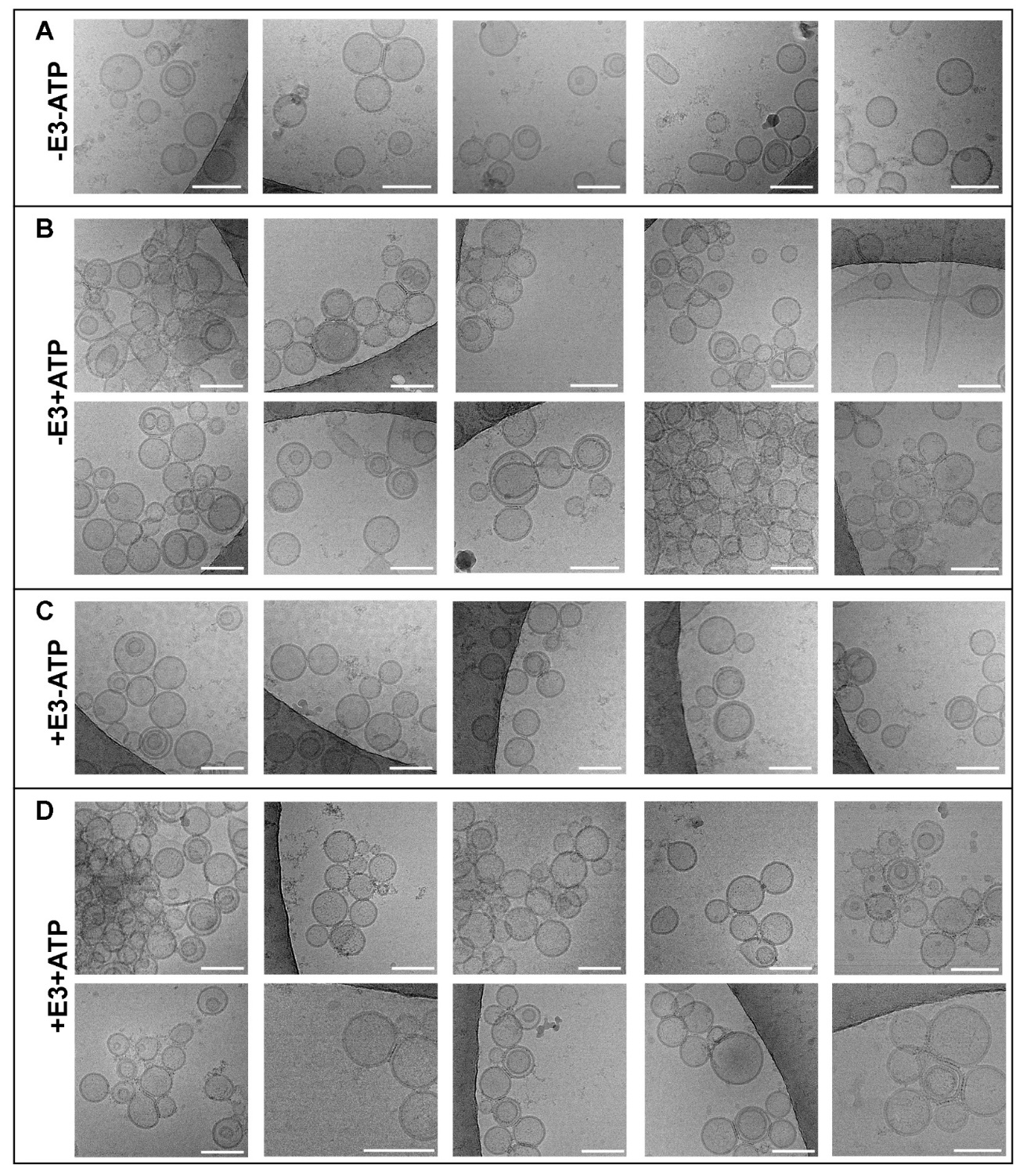


**Supp. Fig. 13.** GABARAPL1 ability to tether and fuse vesicles in the absence and presence of E3 analyzed by cryo-EM. Additional cryo-EM images of the four conditions analyzed in Figure 7. 0.5 µM ATG7, 1 µM ATG3, and 5 µM GABARAPL1 were mixed with 0.4 mM LUVs (ePC:DOPE:PI:DOG (33:55:10:2 mol ratio)), in the absence (-E3) or presence (+E3) of 0.1 µM E3 and, after addition of buffer (-ATP) or ATP (+ATP), they were incubated at 37°C for 90 min. (*A-D*) Cryo-EM images of liposomes after reconstituting GABARAPL1 conjugation reaction. **a** In the absence of E3 and ATP. **b** In the absence of E3 but in the presence of ATP. **c** In the presence of E3 but in the absence of ATP. **d** In the presence of both E3 and ATP. Bar = 100 nm.

**Supp. Table 1.** List of protein constructs used in this work: vector, expression system, detailed protein encoded, and reference study


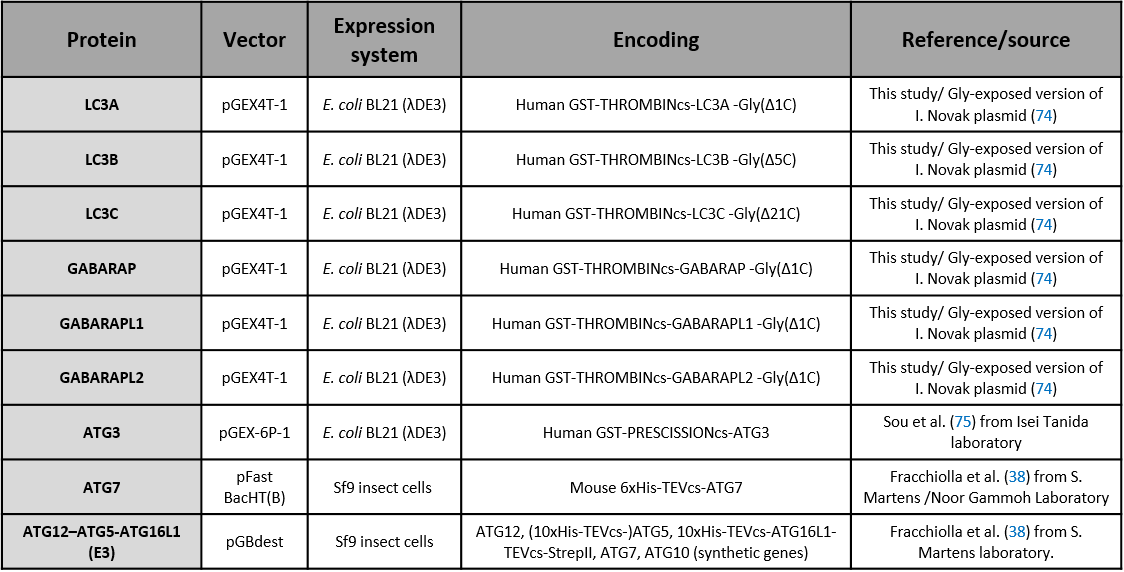

Supplement: Supplementary file 1 — Supplementary file1 (DOCX 3727 kb) [file 18_2023_4704_MOESM1_ESM.docx]
